# Platinum(iv)-azido monocarboxylato complexes are photocytotoxic under irradiation with visible light†

**DOI:** 10.1039/d1dt01730f

**Published:** 2021-07-12

**Authors:** Evyenia Shaili, Marίa J. Romero, Luca Salassa, Julie A. Woods, Jennifer S. Butler, Isolda Romero-Canelón, Guy Clarkson, Abraha Habtemariam, Peter J. Sadler, Nicola J. Farrer

**Affiliations:** Department of Chemistry, University of Warwick Gibbet Hill Road Coventry CV4 7AL UK p.j.sadler@warwick.ac.uk; Departamento de Didácticas Aplicadas, Facultade de Formación do Profesorado, Universidade de Santiago de Compostela 27002 Lugo Spain; Donostia International Physics Center Paseo Manuel de Lardizabal 4 Donostia 20018 Spain; Polimero eta Material Aurreratuak: Fisika, Kimika eta Teknologia, Kimika Fakultatea, Euskal Herriko Unibertsitatea UPV/EHU Paseo Manuel de Lardizabal 3 Donostia 20018 Spain; Ikerbasque, Basque Foundation for Science Bilbao 48011 Spain; Photobiology Unit, Department of Dermatology and Photobiology, Ninewells Hospital Dundee DD1 9SY UK; School of Pharmacy, Institute of Clinical Sciences, College of Medical and Dental Sciences, Sir Robert Aitken Institute for Medical Research, University of Birmingham Birmingham B15 2TT UK; Chemistry Research Laboratory, University of Oxford 12 Mansfield Road Oxford OX1 3TA UK Nicola.Farrer@chem.ox.ac.uk +44 (0)1865 285131

## Abstract

Complexes *trans,trans,trans*-[Pt(N_3_)_2_(OH)(OCOR)(py)_2_] where py = pyridine and where OCOR = succinate (**1**); 4-oxo-4-propoxybutanoate (**2**) and *N*-methylisatoate (**3**) have been synthesized by derivation of *trans,trans,trans*-[Pt(OH)_2_(N_3_)_2_(py)_2_] (**4**) and characterised by NMR and EPR spectroscopy, ESI-MS and X-ray crystallography. Irradiation of **1–3** with green (517 nm) light initiated photoreduction to Pt(ii) and release of the axial ligands at a 3-fold faster rate than for **4**. TD-DFT calculations showed dissociative transitions at longer wavelengths for **1** compared to **4**. Complexes **1** and **2** showed greater photocytotoxicity than **4** when irradiated with 420 nm light (A2780 cell line IC_50_ values: 2.7 and 3.7 μM) and complex **2** was particularly active towards the cisplatin-resistant cell line A2780cis (IC_50_ 3.7 μM). Unlike **4**, complexes **1–3** were phototoxic under green light irradiation (517 nm), with minimal toxicity in the dark. A p*K*_a_(H_2_O) of 5.13 for the free carboxylate group was determined for **1**, corresponding to an overall negative charge during biological experiments, which crucially, did not appear to impede cellular accumulation and photocytotoxicity.

## Abstract 2

Axially-derived platinum(iv) diazido dihydroxido complexes retain dark stability with rapid photoreduction under irradiation with green (517 ± 27 nm) light. Cell uptake is unhindered by overall negative charge at physiological pH (complex **1**).

## Graphical abstract

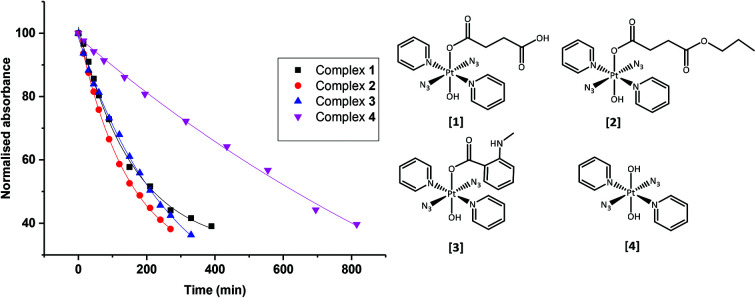

## Introduction

Approximately 40% of the cancer patients treated with chemotherapy receive a platinum(ii)-based medicine such as cisplatin, carboplatin or oxaliplatin.^1^ Despite the wide-spread use of platinum-based drugs^2^ – particularly in combination therapies – disadvantages exist, including the development of resistance and serious side-effects of treatment.^3^ Octahedral, Pt(iv) prodrugs with a d^6^ electronic configuration typically demonstrate greater kinetic inertness than Pt(ii) complexes, as well as offering additional ligand sites for derivation.^4^

Pt(iv) prodrugs are classically regarded to exert their cytotoxic effect following reduction to Pt(ii) species *in vivo*, although Pt(iv) complexes may also form adducts with biomolecules.^5^ Photoactivated chemotherapy (PACT) provides both spatial and temporal control over Pt(iv) prodrug reduction. Photoreduction of Pt(iv) prodrugs of carboplatin,^6^ oxaliplatin^7^ and cisplatin^8^ – either by direct irradiation of the Pt(iv) complex, or by employing separate photosensitisers – have been reported. The ligands coordinated to the Pt(iv) prodrug influence the solubility, lipophilicity and ease of reduction of the complex, whilst the ligands themselves have the potential to be biologically active, through mechanisms which are distinct from the resultant platinum species, following dissociation. The reduction of Pt(iv) complexes is greatly affected by the coordinated ligands; for a series of complexes with chlorido, hydroxido and carboxylato anionic axial ligands [Pt(en)Cl_2_(X)_2_] (where X = axial ligand), cathodic reduction potentials varied by more than 650 mV, with the rates of reduction following the order X = Cl^−^ > OC(O)CH_3_^−^ > OH^−^.^5^ It is notable that for several Pt(iv) complexes – including Satraplatin (*ctc*-[Pt(NH_3_)(CHA)(OAc)_2_Cl_2_] where CHA = cyclohexylamine) – equatorial anionic ligand hydrolysis can occur more readily than axial anionic ligand hydrolysis.^9^ The most important biological reductants, the mechanism(s) and the rate of Pt(iv) reduction in a biological environment for a given complex cannot readily be predicted from the reduction potential.^10^ A range of Pt(iv) complexes undergo photoreduction, typically requiring short wavelengths of light *i.e.* UVA.^11^ The coordination of azido (N_3_) ligands to Pt(iv) results in highly photosensitive complexes, which can be kinetically inert in the dark and – depending on judicious ligand choice – can be photoactivated with longer (visible) wavelengths of light.^12^ Penetration of light into tissue is wavelength-dependent, and extending the wavelength of light which can initiate photoreaction of Pt(iv) complexes – whilst retaining stability in the dark – is therefore a key goal. Replacing the axial OH ligands of Pt(iv) diazido complexes with acetato (OAc) ligands has been demonstrated by TD-DFT to lower the energy of the LUMO and LUMO+1, reducing the energy gap between the HOMO and LUMO.^13^ This has the effect of extending the strongest absorption band to longer wavelengths. Since this modification also has the potential to lead to more facile spontaneous reduction in the dark, evaluation of stability of the complex in the absence of irradiation is important. Modification of the axial ligands of photoactivatable Pt(iv) diazido complexes to incorporate biotin,^14^ guanidinoneomycin,^15^ coumarins^16^ and naphthalimides,^17^ as well as an RGD peptide to target integrin^18^ have been reported. However, the synthesis and extensive characterisation of **1** itself has not been reported previously, and we report it here for the first time along with two other complexes. Hydroxido groups are common Pt(iv) axial ligands because oxidation of Pt(ii) to Pt(iv) is readily accomplished through the use of H_2_O_2_ and also due to the favourable solubility and stability properties imparted by inclusion of the hydroxido ligand. Derivatisation of one hydroxido ligand of a dihydroxido Pt(iv) complex to a carboxylate can be achieved through a number of methods^19^ including reaction with anhydrides,^20–23^ acid chlorides,^24^ and reaction with carboxylic acids in acetic acid solvent.^25^ Terminal carboxylic acids which have been produced through reaction with anhydrides can be used to introduce targeting peptides.^26^ Other functional groups, such as carbamates can also be axially introduced.^27,28^

The aim of the present work was to explore the effect of axial ligand modification on the photochemistry and photobiology of platinum(iv) azido complexes.^12^

**Fig. 1:**
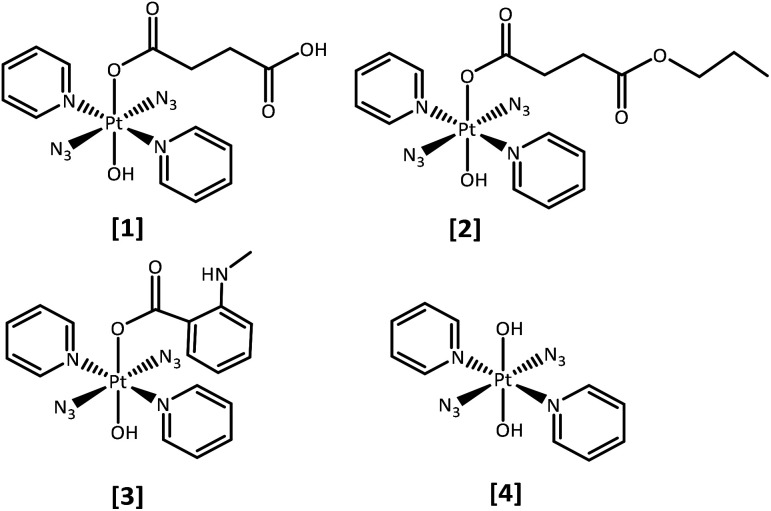
Platinum(iv) azido complexes reported here (axial group): **1** (succinate), **2** (4-oxo-4-propoxybutanoate), **3** (*N*-methylisatoate), and **4** (hydroxide, previously reported).^29^

We report the synthesis, characterisation, photochemical and photobiological studies of the mono-carboxylato compounds: *trans,trans,trans*-[Pt(N_3_)_2_(OH)(succ)(py)_2_] (**1**) where succ = succinate and py = pyridine; *trans,trans,trans*-[Pt(N_3_)_2_(OH)(succ-Pr)(py)_2_] where succ-Pr = 4-oxo-4-propoxybutanoate (**2**) and *trans,trans,trans*-[Pt(N_3_)_2_(OH)(*N*-MI)(py)_2_] where *N*-MI = *N-N*-methylisatoate (**3**), in comparison with *trans,trans,trans*-[Pt(N_3_)_2_(OH)_2_(py)_2_] (**4**), which we have reported previously^29^ (Fig. 1). The *N*-MI ligand was chosen since it introduces an additional absorption band (*ca.* 350 nm) and increased absorption towards longer wavelengths. We therefore anticipated that complex (**3**) might display increased photosensitivity on irradiation with visible light.

## Results

### Synthesis and characterisation

Precursor compounds *N*-methylisatoic acid (*N*-MIA) (**5**), *trans*-[PtCl_2_(py)_2_] (**6**), *trans*-[Pt(N_3_)_2_(py)_2_] (**7**) and complex **4** were synthesized as reported previously; characterisation was consistent with previous reports.^29^ Complexes **1–3** were synthesised from **4** and characterised by ^1^H, ^13^C, ^195^Pt-NMR spectroscopy, ESI-MS and UV-vis spectroscopy (ESI) and single crystal X-ray diffraction.

### X-ray diffraction

**Fig. 2:**
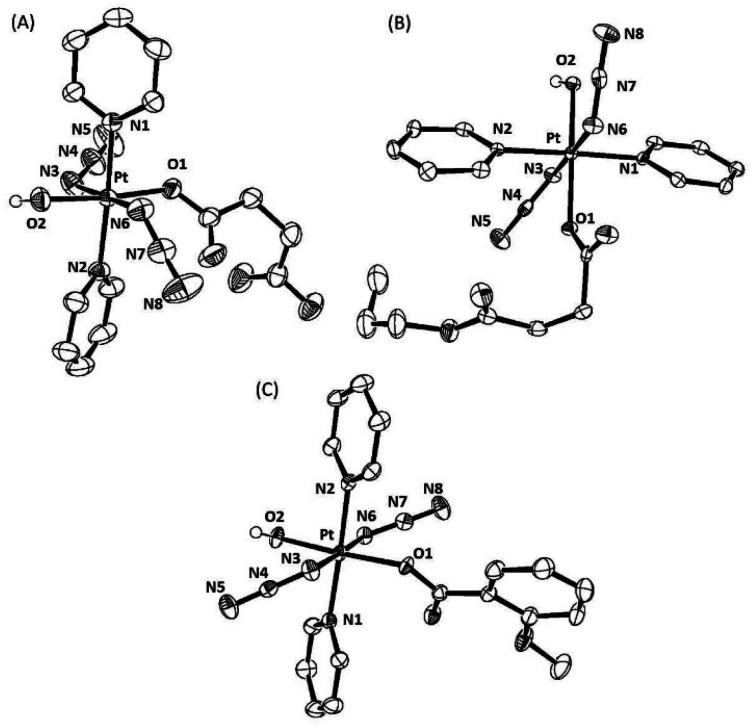
ORTEP diagrams of the complexes: (A) *trans,trans,trans*-[Pt(N_3_)_2_(OH)(succ)(py)_2_] **1**; (B) *trans,trans,trans*-[Pt(N_3_)_2_(OH)(succ-Pr)(py)_2_] **2**; and (C) *trans,trans,trans*-[Pt(N_3_)_2_(OH)(*N*-MI)(py)_2_] **3**. The ellipsoids are set to 50% probability level. Atom labels do not represent the numbering in the cif files (CCDC1998067(**1**); CCDC1998069 (**2**) and CCDC1998068 (**3**)†) but are used to facilitate comparison between the complexes. All hydrogen atoms have been omitted for clarity, except for the axial hydroxido hydrogen atoms.

Single crystals suitable for X-ray diffraction were obtained for complexes *trans,trans,trans*-[Pt(N_3_)_2_(OH)(succ)(py)_2_] **1**, *trans,trans,trans*-[Pt(N_3_)_2_(OH)(succ-Pr)(py)_2_] **2** and *trans,trans,trans*-[Pt(N_3_)_2_(OH)(*N*-MI)(py)_2_] **3** as described in the Experimental section. ORTEP diagrams for the molecular structures are shown in Fig. 2 and the crystallographic data, including selected bond distances and angles are summarized in Table S2.†

Compounds **1**, **2** and **3** show a distorted octahedral geometry around the Pt(iv) ion and differ only in the nature of one of the oxygen donor ligands coordinated in an axial position. The equatorial plane is defined by four nitrogen atoms from two pyridine molecules *trans* to each other (N1 and N2) and two azido anions *trans* (N3 and N6), with an oxygen atom (O2) from a hydroxido ligand located in one axial position. The sixth position is occupied by an oxygen atom (O1) from a monoanionic ligand: a succinate anion in **1**, a 4-oxo-4-propoxybutanoate anion in **2** and a *N*-methylisatoate anion in the case of **3**. The distortion from octahedral geometry is demonstrated by the O2–Pt–O1 bond angle being 174.21(7) Å in **1**, 175.52(9) Å in **2** and 176.50(11) Å in **3**, which are all considerably less linear than the corresponding angle in complex **4** at 179.58(11) Å. The Pt–O1 (carboxylato) bond lengths for the monocarboxylato, monohydroxido complexes **1–3** are all elongated in comparison to **4** and similar to values for previously reported dicarboxylato complexes *trans,trans,trans*-[Pt(N_3_)_2_(OAc)_2_(py)_2_] (2.007(5) Å)^30^ and *trans,trans,trans*-[Pt(N_3_)_2_(CF_3_COO)_2_(py)_2_] (2.0093(15) Å).^15^ In complexes **1–3** the Pt–O1 (carboxyl) bond is longer than the Pt–O2 (hydroxido) bond. However, a difference between both axial Pt–OH bonds was also observed in the crystallographic structure of **4** (Pt–O1 1.990(3) Å and Pt–O2 2.027(3) Å). In **4**, one of the hydroxido ligands is involved in two hydrogen bonds (as a donor and acceptor) with hydroxido and azido groups of a neighbouring molecule, which correlates with the lengthened Pt–OH bond, whereas the second hydroxido ligand acts only as a donor. The intermolecular interactions in complexes **1**, **2** and **3** are shown in the ESI (Fig. S2–S4†).

#### NMR spectroscopy

^195^Pt NMR spectroscopic resonances were observed for **1** (D_2_O), **2** (D_2_O) and **3** (DMSO-*d*_6_) at 1059 ppm, 1065 ppm and 990 ppm, respectively, in the expected low field Pt(iv) region. These are comparable with the resonance reported for compound **4** (D_2_O) at 942 ppm.^29^^1^H and ^13^C NMR spectral resonances were assigned in accordance with previously reported complexes (labelling shown in Fig. S13†).

#### ESI-MS

Pt(iv) diazido complexes commonly form a series of sodiated adducts when analysed by ESI-MS.^31^ Complex **1** gave rise to several species, including [M − N_3_]^+^ at 529.0793 *m*/*z*, [M + Na]^+^ at 594.0783 *m*/*z* and [2M + Na]^+^ at 1165.1673 *m*/*z*. Complex **2** was detected as the [M + Na]^+^ adduct at 636.2 *m*/*z*. The formation of [2M + Na]^+^ adducts was less pronounced for complex **3** than for complex **1**; complex **3** was detected predominantly as [M − N_3_]^+^ at 562.1210 *m*/*z*, with other adducts and species including [M + H]^+^ at 605.1338 *m*/*z* (Fig. S1†).

### p*K*_a_ of complex **1**

The 
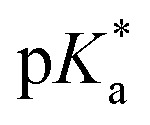
 of complex **1** (1.1 mM in D_2_O) was determined by plotting the change in the ^1^H NMR chemical shift of the pyridine protons against pH*, with dioxane as an internal reference. The pH* was measured at ambient temperature, directly in the NMR tube. The pH* was adjusted to the desired value using DClO_4_. ^1^H NMR spectra were recorded at 25 °C. The data were fitted to the Henderson–Hasselbalch equation (*r*^2^ = 0.99), and the 
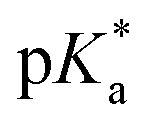
 of the free carboxyl group was determined to be 5.07 (Fig. S5†) in D_2_O, which was converted to a final value of p*K*_a_(H_2_O) 5.13.^32^

### UV-vis spectroscopy

UV-vis spectra of complexes **1–3** (in H_2_O for **1** and **2**; in 5% DMSO/95% H_2_O for **3** due to solubility constraints) were measured, with extinction coefficients determined by ICP-MS analysis of platinum concentration (Fig. S6†).

### DFT and TD-DFT calculations

**Fig. 3:**
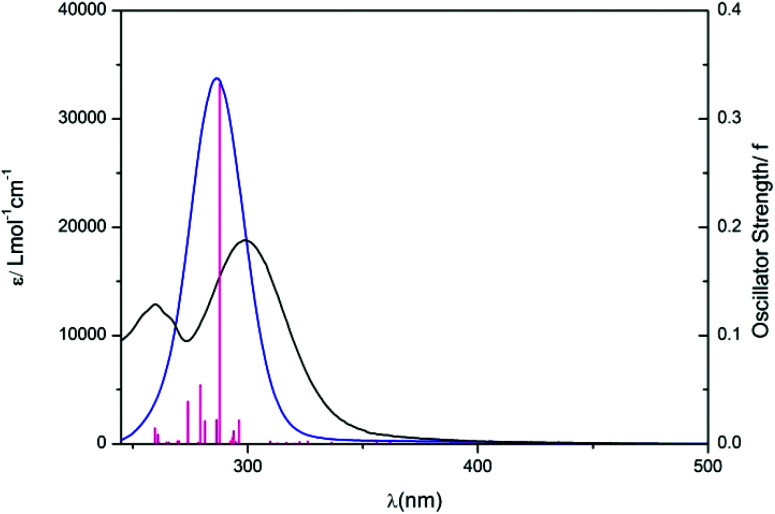
Experimental (black, H_2_O) and theoretical (blue) UV-vis spectra of complex **1** in the deprotonated state. Vertical (red) bars represent the oscillator strengths of the excited states.

DFT and TD-DFT calculations were performed on complex **1** to characterise the electronic transitions giving rise to the UV-vis absorption spectrum and to rationalize the photoactivity of **1**, particularly in comparison to complex **4**.^29^ Ground-state geometry and lowest-lying triplet geometry optimizations were performed in the deprotonated state, since the p*K*_a_ determined (*above*) for **1** (5.13, H_2_O) suggests it would be largely deprotonated at physiological pH. The singlet excited states, as well as the orbital contributions (%) in each case are summarised in Table S3,† along with molecular orbital representations and Electron Difference Density Maps (EDDMS) of singlet excited-state transitions in H_2_O (Table S4†) and molecular orbitals for the ground state (Table S5†). The UV-vis spectra were simulated by calculation of 32 singlet states using water as a solvent (cpcm)^33,34^ and the TD-DFT method (Table 1) and are overlaid with the experimental data in Fig. 3.

**Table 1:** Selected experimental (X-ray crystallography) and calculated bond lengths for complex **1** in both the ground state and the lowest-lying triplet state

Bond	Bond length		
	X-ray structure (Å)	Ground-state (deprotonated)	Lowest lying triplet
Pt–N3(azide)	2.049(2)	2.088	2.359
Pt–N6(azide)	2.045(2)	2.069	2.508
Pt–O2 (OH)	1.9852(15)	2.015	2.015
Pt–O1(CO)	2.0196(16)	2.048	2.065
Pt–N1(py)	2.040(2)	2.068	2.053
Pt–N2(py)	2.032(2)	2.027	2.040
N6–N7	1.206(3)	1.213	1.200
N3–N4	1.202(3)	1.211	1.189
N7–N8	1.137(3)	1.149	1.164
N4–N5	1.126(4)	1.148	1.170

The longest wavelength singlet–singlet transitions in this complex extend up to 469 nm, whereas in **4** they reach to 414 nm.^29^ The transitions S1, S2 and S3 of **1** show that there is a transfer of electron density from the succinate to the platinum centre (^1^LMCT) and also contain ^1^IL (inter-ligand transitions) character. Such transitions (S1, S2, S3) as well as some of the higher energy involve the LUMO, which is antibonding in character with regard to the four equatorial ligands but mainly the azides. Interestingly the LUMO+1, to which electron density is promoted in the higher wavelength transitions (*e.g.* S8, S14) is orthogonal with respect to the LUMO, making it strongly antibonding mostly towards the pyridines and also the hydroxido and the succinate ligand.

This suggests that occupation of the LUMO or LUMO+1 could potentially lead to dissociation of two out of the six ligands. These two ligands would be in a *trans* position to each other. Singlet–singlet transitions in the UV region (*e.g.* S14, S19) occur from lower energy HOMOs and can be assigned as mixed ^1^LMCT/^1^IL. In general, all transitions for complex **1** show some d–d character, as metal orbitals always contribute to the frontier molecular orbitals.

As is commonly observed in DFT calculations,^44–46^ we found that the bond lengths of the ground state of complex **1** are overestimated compared to X-ray, the largest discrepancy being the two Pt–azido bonds, with differences of 0.035 and 0.028 Å (Table 1). In the lowest lying excited-state geometry, Pt–azido bonds are significantly elongated with respect of the ground state (up to 0.44 Å), while variations much less pronounced for the other ligands (Table 1).

### Solution reactivity

Complexes **1–3** showed no signs of decomposition in aqueous solution in the absence of light for at least 1 h at ambient temperature.

### Irradiation studies

#### UV-Vis spectroscopy

**Fig. 4:**
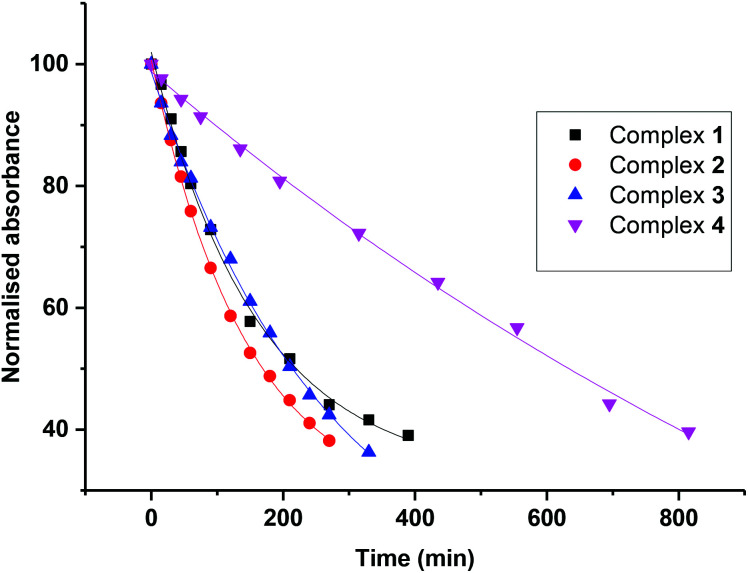
Plot of the decrease in intensity of the N_3_ → Pt LMCT band of compounds **1–4** over time, when irradiated with green light (*λ*_irr_ 517 nm).

Solutions of compounds **1–4** (*ca*. 50 μM, H_2_O) were irradiated with green light (517 nm LED, 29 mW cm^−2^; see Fig. S7† for spectral output of light sources) at ambient temperature, and the photoactivity was monitored by UV-vis spectroscopy (Fig. S8A–D†). The photoactivity of complex **4** has been reported previously,^29^ and it was therefore included here for direct comparison. Consistent with previous reports, a decrease in the intensity of the N_3_ → Pt ligand-to-metal charge-transfer band (the LMCT band in the absorption spectrum of complexes at *ca.* 300 nm) was observed during irradiation of complexes **1–4**, due to dissociation of azido ligand(s). The maximum absorbance for each graph was normalized, such that the first spectrum (*t* = 0 h, prior to irradiation) has a normalized absorbance of 100%. Data points corresponding to the initial data loss of the LMCT band (up to *ca.* 38%) were fitted to an exponential decay and the half-life of each complex was determined (Fig. 4, **4**).^34^ The rates of loss of the LMCT band upon irradiation with 517 nm light ordered from fastest to slowest are (t½, min): complex **2** (169) > complex **3** (214) > complex **1** (220) ≫ complex **4** (634).

Complex **1** was also irradiated with longer wavelength green light (550 nm, filtered below 530 nm with GG530 filter, 4 nm slits, 2.5 mW cm^−2^) to investigate the wavelength limit of photoactivation of the complex (Fig. S8E†). No significant change in the UV-vis spectrum of **1** was observed under these conditions.

#### NMR spectroscopic irradiation studies

**Fig. 5:**
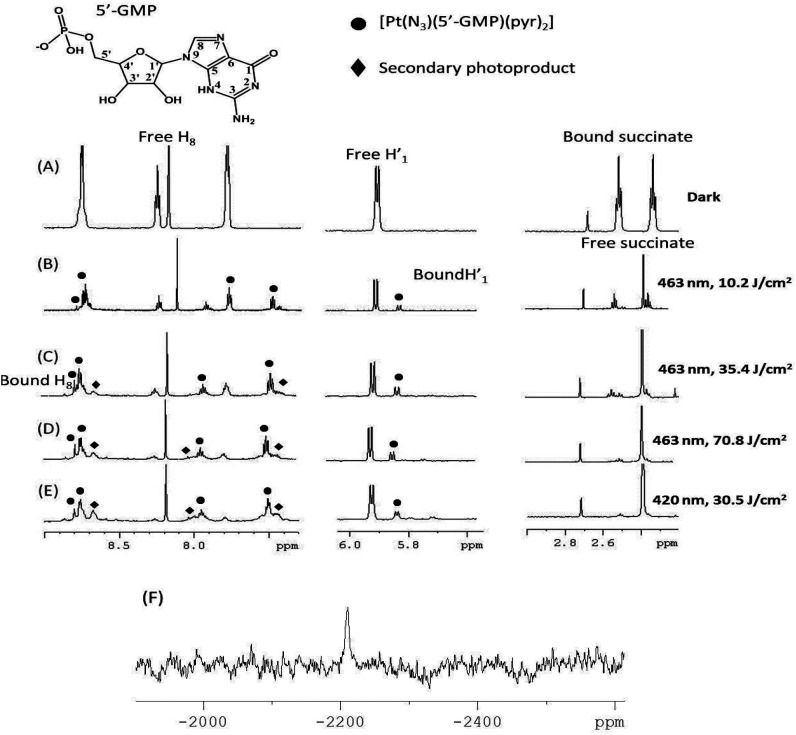
^1^H NMR spectra (500 MHz, PBS in D_2_O, pH 7.4, dioxane) of complex **1** and 5′-GMP (2 mol eq.) under the following conditions: (A) dark; then following irradiation for increasing (total) doses of 463 nm light: (B) 10.2 J cm^−2^; (C) 35.4 J cm^−2^; (D) 70.8 mJ cm^−2^; followed by an additional dose of shorter wavelength (420 nm) light (E) 30.5 J cm^−2^. ● and ◀ denote the two main photoproducts. The sharp singlet at 2.40 ppm corresponds to free succinate. (F) ^195^Pt NMR spectrum (129 MHz) following irradiation, showing resonance at −2210 ppm, assignable to the previously reported *trans*-[Pt(N_3_)(5′-GMP)(py)_2_].^29^

The photoactivity of complex **1** in comparison to **4** was investigated in the presence of the nucleotide guanosine 5′-monophosphate (5′-GMP) by ^1^H NMR spectroscopy. Irradiation (10 min, 463 nm, 64 mW cm^−2^) of **1** resulted in 23% coordinated 5′-GMP in the species *trans*-[Pt(N_3_)(5′-GMP)(py)_2_], compared to only 9% of 5′-GMP coordinated for **1** (Fig. S9†). Under continued irradiation of **1** (463 nm), followed by a final irradiation with shorter wavelength (420 nm) light, the formation of two main photoproducts became evident from the ^1^H NMR spectra (Fig. 5). One of these can be identified as the mono-azido 5′-GMP adduct, *trans*-[Pt(py)_2_(N_3_)(5′-GMP)], with assignment confirmed by LC-MS and ^195^Pt NMR spectroscopy.^29^

The photoactivity of complex **1** was also investigated through monitoring the release of the succinate ligand in the presence and absence of 5′-GMP. Photorelease of the succinate ligand from **1** occurred *ca.* 3× more slowly in the presence of 5′-GMP than in its absence (Fig. S10†). A precipitate was also observed in the absence – but not in the presence – of 5′-GMP, consistent with previous reports.^29^

Due to the limited aqueous solubility of **3**, irradiation studies in the presence of 5′-GMP were carried out in 75% MeOH-*d*_4_/25% D_2_O solution. In contrast to complex **1**, very little 5′-GMP binding was observed for **3** following irradiation, and pyridine release was clearly observed (Fig. S11,† top).

To establish whether the release of pyridine was solvent-dependent, complex **4** was irradiated in 75% *d*_4_-MeOH/25% D_2_O in the presence of 5′-GMP, with either 420 nm or UVA light. Pyridine release was also observed from **4** under these conditions (Fig. S11,† bottom), which was not observed when **4** was irradiated in D_2_O or H_2_O solutions with no MeOH-*d*_4_ present.^29^ The release of the axial *N*-methylisatoate (*N*-MIA) ligand from complex **3** in aqueous solution was also monitored by fluorescence emission spectroscopy (Fig. S12†), upon excitation of complex **3** at 320 nm, which demonstrated an increase in fluorescence consistent with ligand release.

#### LC-MS data

**Fig. 6:**
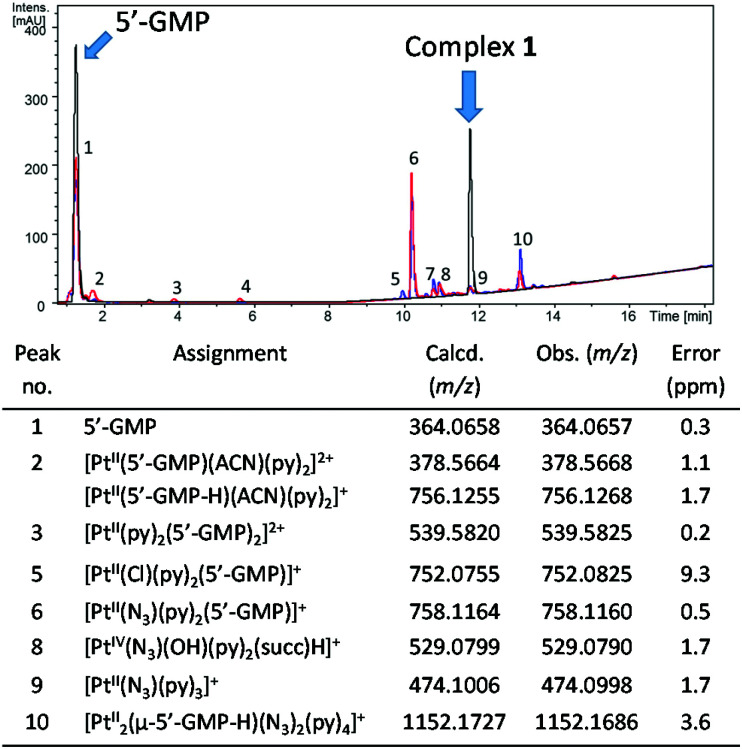
Chromatogram (*λ*_detection_ 240 nm, ACN/H_2_O, 0.1% formic acid) for complex **1** in the dark (black trace) or after irradiation in the presence of 2 mol. eq of 5′-GMP in H_2_O (red trace, **1** (0.5 mM), *λ*_irr_ 420 nm, 30.5 J cm^−2^) and in PBS (blue trace, **1** (1.5 mM) *λ*_irr_ 463 nm, 153.6 J cm^−2^) with identifiable peaks assigned. Corresponding mass spectral data for numbered peaks are given in Table S6.†

LC-MS was employed to characterise the photoproducts formed following irradiation of complex **1** in the presence of 5′-GMP (Fig. 6). For comparison, the mass spectrum of complex **1** in the dark is given in Fig. S1.† Complex **1** was irradiated in the presence of 2 mol. eq. of 5-GMP in either H_2_O (Fig. 6, red trace) or under similar conditions but in PBS (0.140 M [Cl^−^], Fig. 6, blue trace). Comparable adducts were seen under both conditions, including [Pt^II^(N_3_)(py)_2_(5′-GMP)]^+^ (peak 6) and [Pt^II^_2_(μ-5′-GMP-H)(N_3_)_2_(py)_4_]^+^ (peak 10) species, although irradiation in H_2_O resulted in detection of greater amounts of [Pt^II^(5′-GMP)(ACN)(py)_2_]^2+^ and irradiation in the presence of chloride-containing PBS solution lead to the additional formation of [Pt(py)_2_(5′-GMP)Cl]^+^ (peak 5).

**Fig. 7:**
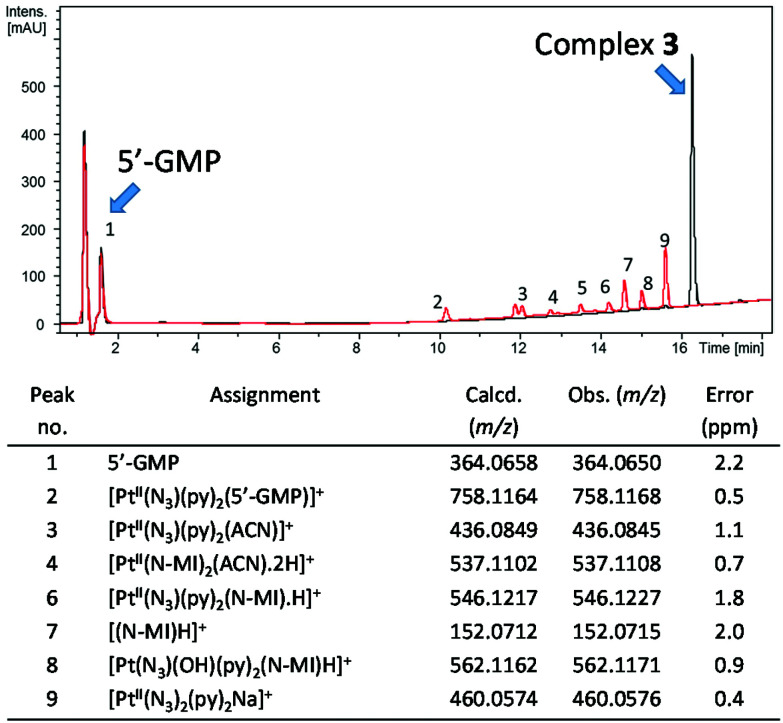
Chromatogram (*λ*_detection_ 240 nm, ACN/H_2_O, 0.1% formic acid) for complex **3** in the dark (black line) and after irradiation with 420 nm light in the presence of 2 mol. eq of 5′-GMP in H_2_O (red line) with identifiable peaks assigned. Corresponding mass spectral data for numbered peaks are given in Table S7.†

The photoproducts from irradiation (*λ*_irr_ 420 nm, 45 min) of complex **3** (1.5 mM) plus 5′-GMP (2 mol. eq) in MeOH : H_2_O (75% : 25%, v/v) were also analysed by LCMS (Fig. 7). In addition to the platinum photoproducts, photoproducts corresponding to loss of pyridine were observed, and free *N*-MI ligand was also detected (peak 7) at 152.0715 *m*/*z*.

### EPR spectroscopy

**Fig. 8:**
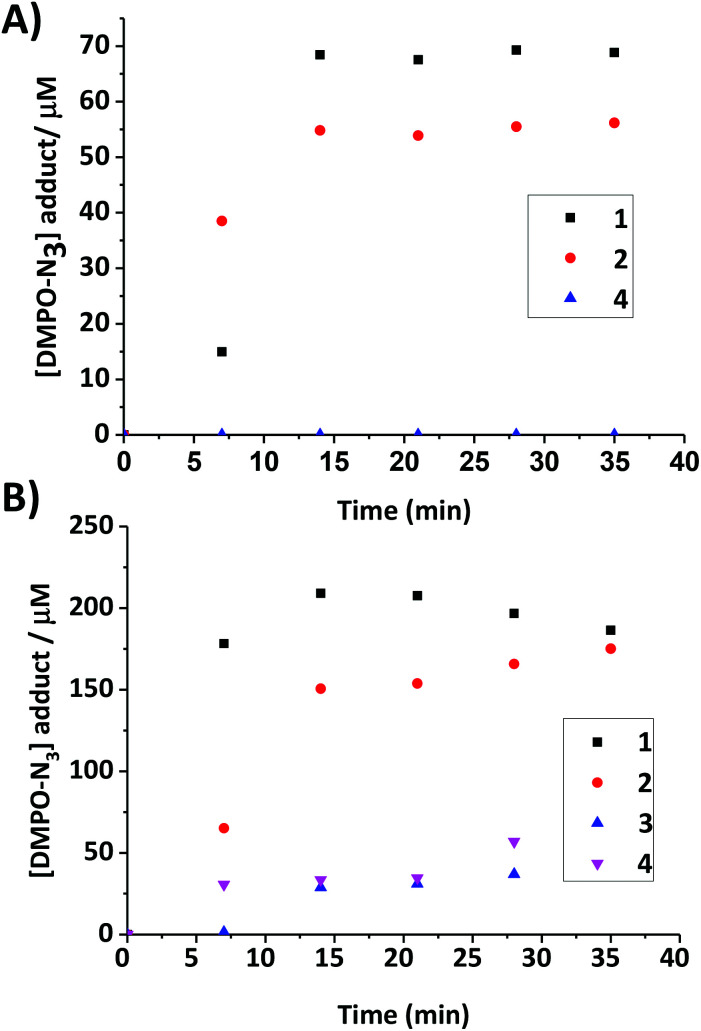
Time dependent trapping of azidyl radicals with DMPO (2 eq.) upon continuous irradiation of complexes with green light (517 nm, 33 mW cm^−2^); (A) complexes **1**, **2** and **4** PBS (1 mM) and (B) complexes **1–4** in DMF/H_2_O (75%/25% v/v, 2 mM).

Compounds **1**, **2** and **4** (1 mM, in PBS) were irradiated with green light (517 nm, 33 mW cm^−2^) in the presence of the spin-trap 5,5-dimethyl-pyrroline *N*-oxide (DMPO) (2 mM). The yield of trapped azidyl radicals (DMPO-N_3_) was determined by the double integration of the EPR signals, quantified by comparison to Tempol (Fig. 8A). EPR spectra of the solutions were also recorded in the dark, during which time no azido radical trapping was observed. Analysis of the DMPO-N_3_ production at regular time-points (0, 7, 21, 28, 35 min) during the irradiation demonstrated that the two compounds with carboxylate ligands (**1** and **2**) produced azido radicals when irradiated with 517 nm light, whereas the dihydroxido compound **4** did not produce an appreciable concentration of azidyl radicals. Complexes **1–4** were then investigated for their ability to generate azidyl radicals in DMF/H_2_O (75%/25% v/v), since it was a suitable solvent system for allowing direct comparison of all complexes.

The yield of azido radicals was determined at regular time-points during irradiation (0, 7, 14, 21, 28, 35 min), following a similar trend to the PBS solution, with compounds **1** and **2** giving rise to more intense EPR signals than compound **4**, which, with the inclusion of DMF, did produce azidyl radicals under green light irradiation.

**Fig. 9:**
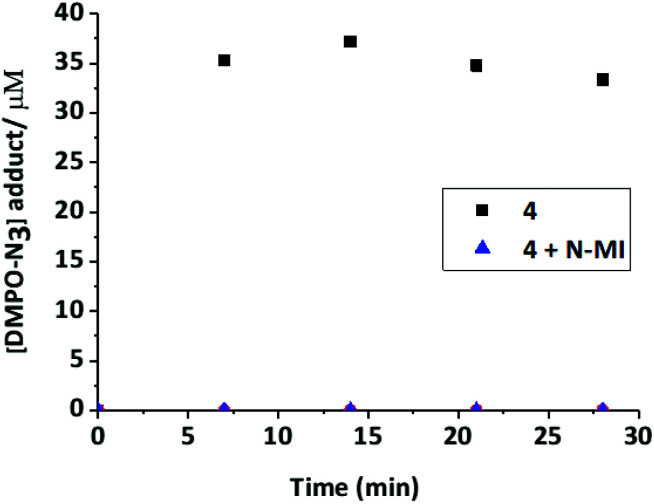
[DMPO-N_3_] signal upon continuous irradiation (463 nm) of complex **4** (2 mM) alone, or in the presence of 2 mol eq. of the *N*-MI ligand in DMF/H_2_O (75%/25%) at ambient temperature.

Compound **3** produced only a very low yield of azidyl radicals (Fig. 8B). To investigate the effect of the *N*-MI ligand on the yield of azidyl radicals, a solution of complex **4** (2 mM, DMF/H_2_O (75%/25% v/v)) was irradiated with shorter wavelength light (blue, 463 nm, 64 mW cm^−2^) in the presence of free *N*-MI (2 mol eq., 4 mM) and DMPO (2 eq., 4 mM) and the azido radical yield was measured at regular time-points. This demonstrated a marked decrease in azido radical trapping in presence of the free ligand compared to in its absence (Fig. 9).

#### Phototoxicity and cell uptake studies

**Fig. 10:**
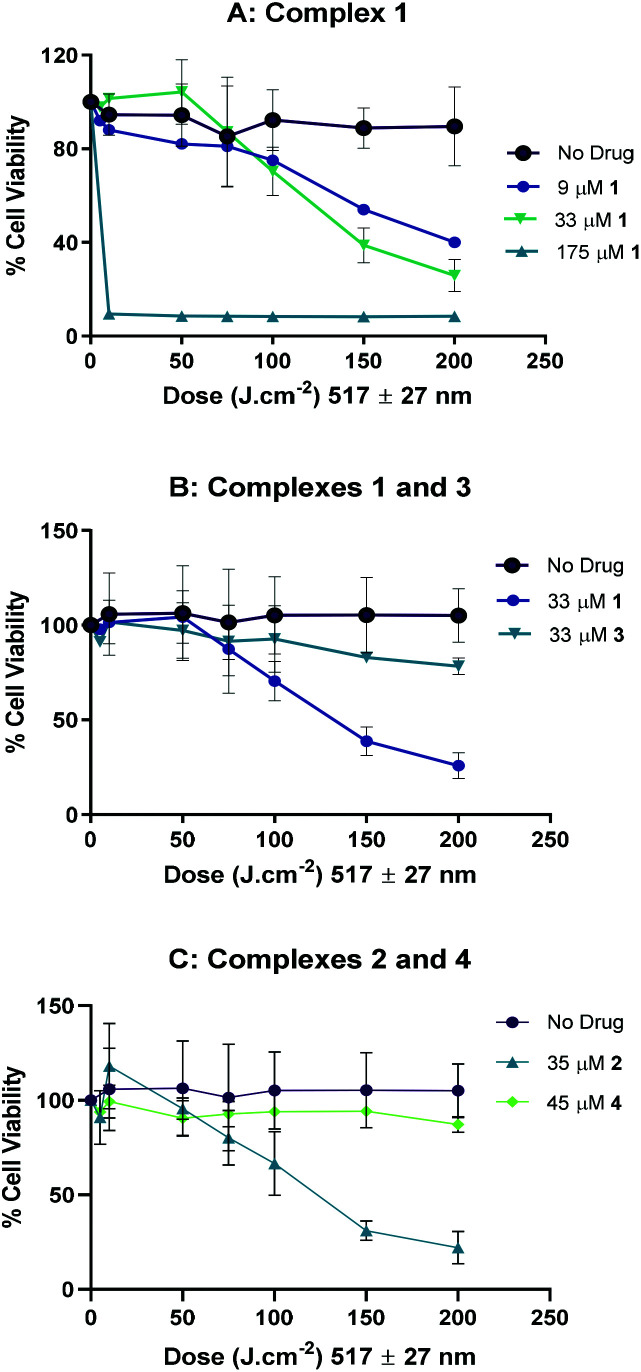
Anti-proliferative activity of complexes **1–4** after photoactivation with green light (517 ± 27 nm) in A2780 cells: (A) in the presence of increasing concentrations of complex **1**; (B) in the presence of complexes **1** or **3** and (C) in the presence of complexes **2** or **4**. Data points represent the mean ± SD of two independent experiments performed in triplicate.

The cytotoxicity and photocytotoxicity of complexes **1–4** (IC_50_ values, Table 2) were determined in three cancer cell lines (A2780 (ovarian), A2780cis (ovarian, cisplatin-resistant), and OE19 (oesophageal) as described in the ESI.† Cells were exposed to the complexes for 1 h in the dark, and then either irradiated or sham-irradiated with blue light (5 J cm^−2^*λ*_max_: 420 nm). IC_50_ values were determined 24 h post-exposure, and photocytotoxicity indices (PI = IC_50_(dark)/IC_50_(light)) were calculated, where a large PI is desirable, indicating low toxicity in the dark and high phototoxicity. In most cases it was not possible to determine an IC_50_ value for the sham-irradiated cells, due to the low toxicity of the complexes in the dark. Instead the mean viability obtained in the dark at the maximum administered dose is given. Cytotoxicity experiments to determine IC_50_ values also compare the percentage of cell viability of untreated cells between the irradiated and dark samples, in all cases there is no statistical difference between these populations. Such controls guarantee that the reduction in cell viability observed in the samples is as a results of the complex activation rather than a light effect. Poorly soluble complex **3** was solubilised in DMSO and diluted into buffered salt solution (0.7% v/v) before administering to the cells. Therefore, it was tested at a maximum of 20 μg ml^−1^ (33 μM) instead of 100 μg ml^−1^. In the absence of a (dark) cytotoxic IC_50_ value, the PI of **3** cannot therefore be directly compared to **1**, **2** and **4**, as it will be artificially lower. Replacement of a hydroxide ligand (**4**) with either a succinate (**1**) or 4-oxo-4-propoxybutanoate (**2**) ligand slightly enhanced the PI for A2780 cells, and increased the PI of the complex in the platinum-resistant A2780cis cell line. Inclusion of either succinate (**1**) or 4-oxo-4-propoxybutanoate (**2**) did not increase the PI in OE19 oesophageal cells, in fact, the 4-oxo-4-propoxybutanoate ligand appeared to decrease it, relative to complex **4**. Complexes **2** and **3** were similarly phototoxic in the platinum-sensitive and platinum-resistant ovarian cancer cell lines, whereas complexes **1** and **4** were slightly less phototoxic in the A2780cis cell line. Complex **3** was the least phototoxic of the four complexes, but was as active against the oesophageal line as towards the ovarian cells. The potential protective effect of tryptophan (a known azido radical scavenger)^35,36^ was investigated; irradiation of complexes **1** and **4** in the presence of 1 mM tryptophan reduced the phototoxicity by 3-fold and 4-fold respectively (Table 3). To investigate the phototoxicity of the complexes under irradiation with longer wavelength light, complex-treated A2780 cells were irradiated with either green (517 ± 27 nm), or yellow (570 ± 27 nm) light, or sham-irradiated from a light guide in stirred solution. Viability was determined 72 h post-exposure, to interrogate anti-proliferative activity. The phototoxicity of complex **1** with green light was investigated in both a complex- and light-dose dependent manner, and it increased with both increasing concentration and light dose (Fig. 10A). Complexes **1** (33 μM) and **2** (35 μM) showed similar phototoxicity under irradiation with 517 nm light; 50% cell viability 72 h post-irradiation was observed at light doses of 112 J cm^−2^ [86–146 J cm^−2^] for **1** (Fig. 10B) and 105 J cm^−2^ [74–150 J cm^−2^] for complex **2** (Fig. 10C).

**Table 2:** Phototoxicity of **1**, **2**, **3** and **4** in human carcinoma cell lines 24 h post-activation with 5 J cm^−2^ visible blue light (*λ*_max_: 420 nm)

Complex	Cell line^a^											
	A2780				A2780cis				OE19			
	IC_50_/μM	95% CI	Viability ± SE at MAD (%)	PI	IC_50_ /μM	95% CI	Viability ± SE at MAD (%)	PI	IC_50_/μM	95% CI	Viability ± SE at MAD (%)	PI
**1**	2.7	2.2–3.4	65.5 ± 13.8	>49	5.4	4.3–6.7	75.4 ± 2.3	>37	8.2	5.3–12.7	89.3 ± 16.4	>21
**2**	3.7	3.0–4.6	62.2 ± 5.1	>44	4.0	2.9–5.4	59.9 ± 6.8	>41	10.3	Wide	74.4 ± 3.5	>15
**3**	13.4	6.0–30.3	79.5 ± 5.6	>2	13.0	6.8–24.6	77.5 ± 1.6	>2	17.5	10.3–29.8	85.5 ± 2.1	>1.9
**4**	5.1	3.7–7.0	98.7 ± 8.4	>41	8.0	5.2–12.3	81.4 ± 5.3	>26	8.4	6.5–10.8	98.6 ± 5.1	>25

a IC_50_: concentration of drug that inhibited dye uptake by 50%. 95% CI: 95% confidence interval of the IC_50_ value. MAD: maximum administered dose. In the absence of a cytotoxic IC_50_ value, the reported PI values are ‘at least’ the value stated (denoted by ‘>’). Results are the mean of 2–5 independent experiments performed in triplicate.

**Table 3:** Phototoxicity of **1** and **4** in A2780 carcinoma cells 24 h post-activation with 5 J cm^−2^ visible blue light (*λ*_max_: 420 nm) in the presence of 1 mM l-tryptophan

Complex	Without tryptophan^a^		With 1 mM tryptophan^a^		Protection factor
	IC_50_ (μM)	95% CI	IC_50_ (μM)	95% CI	
**1**	2.4	2.1–2.7	7.3	2.3–23.3	3.0
**4**	5.3	3.1–9.2	22.5	7.6–67.0	4.2

a IC_50_: concentration of drug that inhibited dye uptake by 50%. 95% CI: 95% confidence interval of the IC_50_ value. Results are the mean of 2 independent experiments performed in triplicate.

Complexes **3** (33 μM) and **4** (45 μM) demonstrated negligible photoactivity under these conditions (Fig. 10B and C respectively). No reduction in cell viability by complex **1** was observed under irradiation with even longer-wavelength light (570 ± 27 nm, 175 μM complex **1**: Fig. S14†). These experiments also demonstrate the lack of dark toxicity of the complexes compared to the control cells treated with light only.

**Fig. 11:**
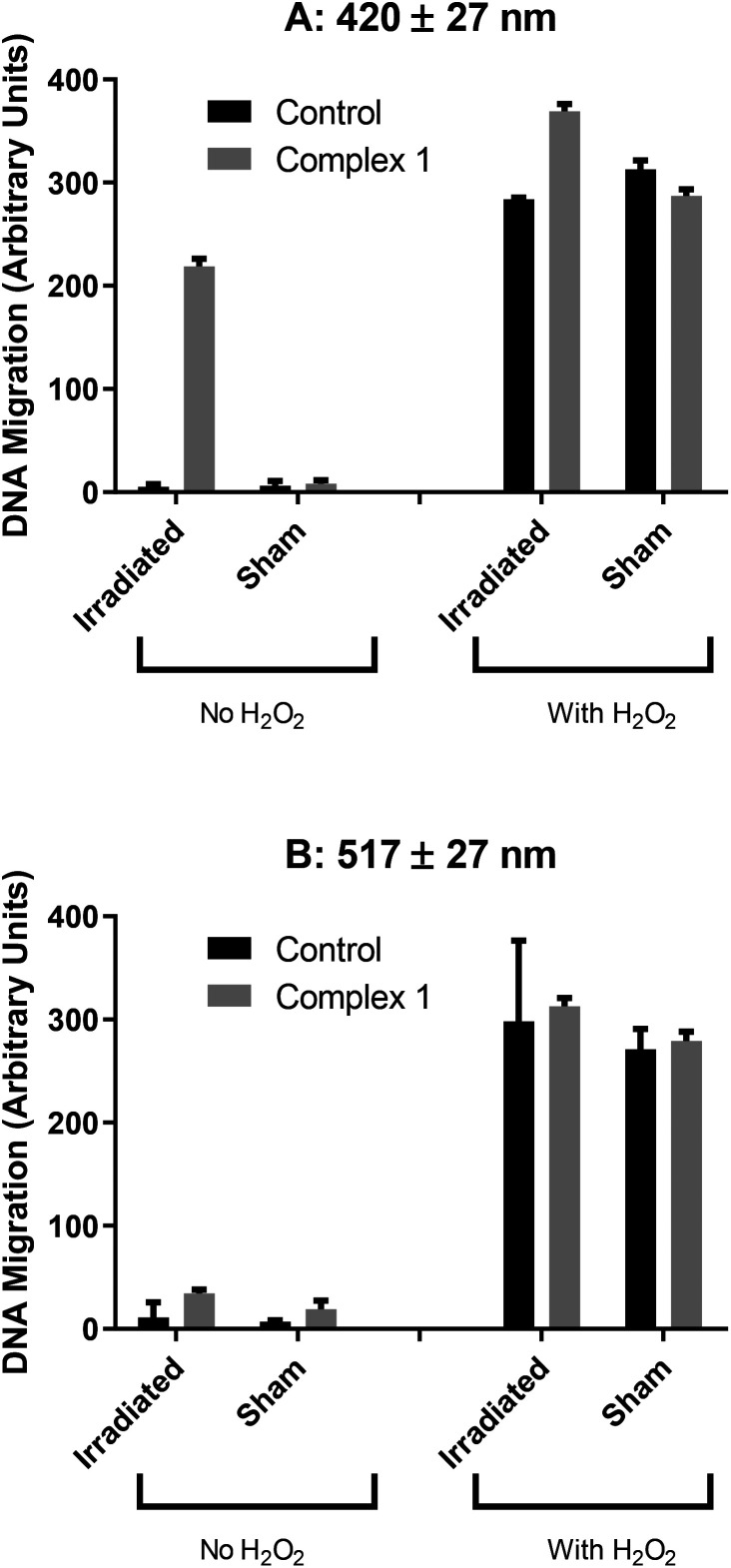
DNA migration in A2780 cells following photoactivation of complex 1 (35 μM) with 10 J cm^−2^ of either (A) blue 420 nm or (B) green 517 nm light.

The phototoxicity of complex **1** correlated with the results of a comet assay (which was not performed with complex **2** as well, due to time constraints), which showed increased DNA migration when A2780 cells were irradiated (100 J cm^−2^ 420 nm light) in the presence of the drug (Fig. 11A). This increased migration might be due to reactive species produced on photoactivation, but is more likely due to cell death, as this combination of drug and light is cytotoxic. DNA migration was not increased when the same concentration of drug was activated with longer-wavelength (100 J cm^−2^ 517 nm) light (Fig. 11B) which could be due to reduced toxicity at this wavelength (*ca.* 50% cell death) or the nature of the reactive species produced.

### Cellular uptake studies

**Fig. 12:**
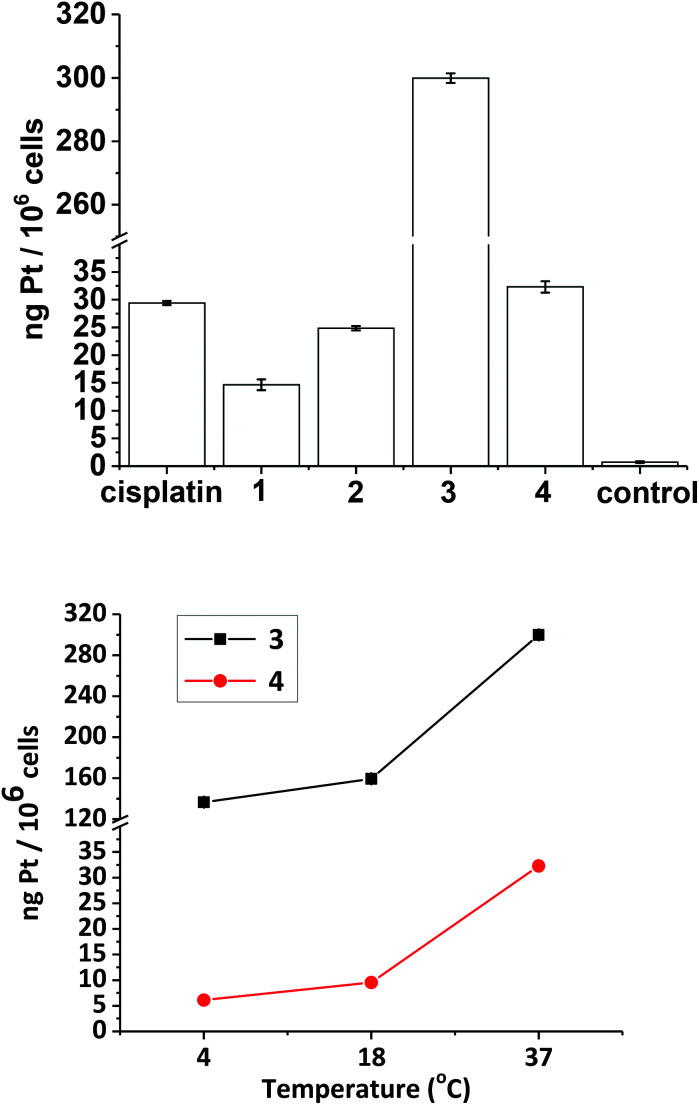
Top: cellular uptake studies of compounds **1–4** (ng Pt/10^6^ cells) at 37 °C with cisplatin for comparison. Bottom: temperature-dependence on the cellular uptake of complexes **3** and **4**, after 1 h drug incubation in the dark. Each experimental condition was carried out in triplicate in the A2780 human ovarian cancer cell line.

Cellular uptake studies were carried out for complexes **1–4** with the established Pt(ii) anti-cancer complex cisplatin as a control, in the A2780 cell line. The concentration used for the uptake of all of the complexes was 20 μM, since no dark toxicity was observed at this concentration for any of the compounds. Uptake of Pt from the compounds at 37 °C followed the order (from highest to lowest): **3** (300 ng Pt/10^6^ cells) ≫ **4** (32 ng Pt/10^6^ cells) > cisplatin (29 ng Pt/10^6^ cells) > **2** (25 ng Pt/10^6^ cells) > **1** (15 ng Pt/10^6^ cells) (Fig. 12, top). The temperature-dependence of cellular uptake was studied by measuring uptake at 4 °C, 18 °C and 37 °C (Fig. 12, bottom) for compounds **3** and **4**. Both compounds followed the same trend, with uptake increasing with increasing temperature.

## Discussion

### Synthesis and characterisation

*trans*-Dihydroxido Pt(iv) complexes can be axially functionalised through use of anhydrides, acid chlorides, pyrocarbonates, and isocyanates to form carboxylates, carbonates and carbamates, respectively.^27,37–39^ Three novel complexes **1–3** have been synthesised, axial ligation was achieved through reaction of **4** with the corresponding anhydride (either succinic anhydride or *N*-methylisatoic anhydride) through a ring-opening reaction. Complex **2** was then obtained from **1***via* esterification of the succinate ligand with sodium propanolate, resulting in formation of a 4-oxo-4-propoxybutanoate group. Incorporation of the *N*-methylisatoate ligand in complex **3** was chosen as it was anticipated that it could impart greater photosensitivity to the complex.

### NMR spectroscopy and X-ray crystallography

The higher ^195^Pt NMR chemical shifts (D_2_O) for complexes **1** (1059 ppm) and **2** (1065 ppm) in comparison to **4** (942 ppm) indicate that the ^195^Pt nucleus is more deshielded following carboxylation; complex **3** was measured in DMSO-*d*_6_ due to solubility constraints and therefore a direct comparison for **3** is less helpful. Lengthening of the Pt–P bond in the case of platinum complexes containing phosphine ligands has been reported to increase the ^195^Pt NMR chemical shift.^40^ Here, the Pt–O bonds to carboxylate ligands are systematically marginally longer than the Pt–OH bond for **1–3** (Table S2†), which may account for the increase in the chemical shift.

Activation of prodrugs with longer-wavelength light is desirable to achieve greater penetration into tissues. Therefore, the longer wavelength absorption bands in the UV-vis spectra are of particular interest for photoactivatable metallodrugs. Green light has greater clinical relevance, penetrating *ca.* 50% more deeply into tissues than shorter wavelength blue light. Complexes **1–4** show two absorption bands at *ca.* 260 nm and 300 nm (Fig. S6 and S8†), composed mainly of transitions with ^1^LMCT character. The absorption bands in compounds **1–3** are red-shifted in comparison with those in the precursor **4**, indicating that modification of the axial group has the potential to enhance the photolability of the Pt(iv)-diazido prodrugs when irradiated at longer wavelengths. The nature of the axial ligand also affects the pharmacological properties of the drugs, such as lipophilicity, water solubility and stability. Biological reducing agents in the cell and in the blood plasma include ascorbate, glutathione as well as cysteine-rich metallothioneins.^41^ Glutathione levels vary widely, but in healthy adults, mean GSH concentrations are reported as 849 ± 63 μM in whole blood and 3.39 ± 1.04 μM in blood plasma.^42^ Intracellular levels are typically higher, around 2–8 mM in the cytosol.^43^ Compared to disease-free tissue, glutathione levels tend to be elevated in ovarian tumours.^44^ It is known that complexes with axial acetate ligands have the potential to be more readily reduced than those with hydroxido ligands: the monocarboxylato Pt(iv) analogue of oxoplatin (*cis,trans,cis*-[PtCl_2_(OH)_2_(NH_3_)_2_]) is reduced faster in the presence of glutathione than oxoplatin.^41^ We determined that **1** showed good stability in the presence of 2 mol eq. of GSH, which is important for its potential development as a photoactivatable Pt(iv) prodrug, since these need to be relatively stable in a biological environments following administration before being activated by irradiation.

### DFT and TD-DFT

The theoretical UV-vis maximum appears at 286 nm (33 748 mol^−1^ cm^−1^) compared to the experimental maximum at 299 nm (*ε* = 18 796 mol^−1^ cm^−1^). TD-DFT calculations therefore slightly underestimate the absorption maximum by *ca.* 10 nm and overestimate the value of the extinction coefficient. The method used for these TD-DFT calculations is the same as that used for **4**, for which a closer match between the theoretical and the experimental extinction coefficient was found.^30^ The larger difference between calculation and experiment for **1** is therefore ascribed to the inclusion of the succinate ligand. Although functional and basis set benchmarking testing might improve the agreement between calculated and experimental transitions, the results obtained are still satisfactory to characterise the nature of the UV-Vis absorption bands. Overall, our results show that light excitation would results in the formation of low-energy singlet states of mixed nature, in which the strong ^1^LMCT component and the nature of the LUMOs involved is consistent with a variability in the photoproducts.

Besides, transition metal complexes such as d^6^-Pt(iv) derivatives usually undergo intersystem crossing (IC) to triplet states upon light-excitation. Therefore, the nature of the lowest-lying triplet state is crucial to understand their photochemical behavior, since such a state is significantly populated before the molecule relaxes to the ground state. Elongation of bonds in the triplet state indicates that they could break upon photoexcitation, and therefore suggests which ligands are most likely to dissociate. The difference between the lowest-lying excited-state geometry and the ground state demonstrates that for complex **1**, both Pt-azido bonds show the biggest elongation, the Pt–OH bond does not elongate, the Pt-succinate bond is elongated, and one of the Pt-pyridine bonds is elongated whereas the other is shortened (Table S2†). This is consistent with the experimental observations which include release of the succinate ligand (Fig. 5) and azido radicals (Fig. 8).

### Solution reactivity

Photoreductions of **1–4** were monitored by UV-vis spectroscopy (spectral output of the light sources are given in Fig. S7†), showing a decrease of the N_3_ → Pt ligand–to–metal charge-transfer (LMCT) band (at *ca.* 300 nm) due to the loss of an azido ligand(s) (Fig. S8†), following an exponential decay profile (Fig. 4, **4**). The photoreduction occurred faster for the monocarboxylato complexes **1–3** than for the hydroxido complex **4**, indicating that the carboxylate functionality enhances the photoreactivity of these complexes. Furthermore, complex **2**, showing the fastest photodegradation kinetics, shows the longest Pt–O (carboxylate) bond, which photodissociates upon irradiation. The increased rate of photodegradation for the monocarboxylate complexes correlates with the extinction coefficient, which is higher at longer wavelengths (Fig. S6†).

### EPR spectroscopy

The formation of azidyl radicals when Pt(iv) diazido complexes are irradiated is thought to play a key role in the mechanism of action of these prodrugs.^35^ Therefore the photoproduction of azidyl radicals from **1–4** was studied by EPR spectroscopy. Under green (517 nm) light irradiation, PBS solutions of complex **4** did not generate trapped radicals, whereas **1** and **2** produced radicals at concentrations up to 68 μM and 55 μM, respectively. The identity of the trapped radical was confirmed as DMPO-N_3_ by matching the hyperfine splitting constants (HFSC) to the theoretical data reported previously.^47,48^ Hydroxyl or carboxylate radicals, which could potentially be released from the Pt(iv) complex, would give a 1 : 2 : 2 : 1 pattern when trapped by DMPO; this signal was not detected in the spectrum. Initial production of radicals (after 7 min of irradiation) was lower for **1** than for **2**, although the azidyl radical yield after 14 min of irradiation was slightly higher for **1** than for **2**. Both complexes **1** and **2** gave rise to significantly greater azido radical production under green light irradiation than the parent dihydroxido complex **4**. A similar experiment was carried out in DMF/H_2_O (75%/25% v/v) to enable comparison between all four complexes and to investigate the effect of solvent. Compounds **1**, **2** and **4** in DMF/H_2_O (75%/25% v/v) gave broadly similar results as seen in PBS, following prolonged irradiation, with **1** giving the highest and **4** the lowest azidyl radical yield, although at the first measurement (7 min) in DMF/H_2_O **1** showed greater azide radical production than **2**, whereas **2** had showed greater initial azide radical production than **1** in PBS. Irradiation of compounds **3** and **4** resulted in a similar, although modest, concentration of azide radicals being trapped (which for **4** was higher than the radical production observed in PBS), despite the rate of photodegradation of **3** being ∼3-fold higher than that of **1** (Fig. 4). The *N*-methylisatoate ligand (axially bound in **3**) may act as a radical scavenger, which could explain the low levels of azido radical trapping for **3**. In order to confirm this hypothesis, complex **4** was irradiated (*λ* = 463 nm, 64 mW cm^−2^) in the presence of *N*-methylisatoic acid (2 mol eq.). The production of azidyl radicals was entirely quenched by the presence of *N*-methylisatoate acid, suggesting that upon release, this ligand may readily react with N_3_˙ Fig. 9. Methyl anthranilate, which is structurally related to the *N*-methylisatoate ligand, has been shown to quench singlet oxygen due to the presence of the anilinic moiety.^49^

### Biological data

The phototoxicity of complexes **1**, **2** and **4** towards A2780 cells after irradiation with green 517 nm light did not correlate with levels of Pt uptake by the cells (Fig. 12), but did correlate with azidyl radical production in PBS (Fig. 8), implicating the azide radicals in the phototoxic mechanism of action of the complexes. If the azide radicals are scavenged by the *N*-methylsatoate ligand this could explain the lower relative phototoxicity of complex **3**, despite cellular uptake studies demonstrating a Pt content in A2780 cells for **3** at least 10-fold that detected for **1**, **2**, and **4**. Furthermore, co-incubating A2780 cells with 1 mM tryptophan protected against blue-light induced phototoxicity in cells which had been treated with either **1** or **4**, with slightly more protection afforded to the cells treated with **4**. Tryptophan is an efficient scavenger of singlet oxygen^50^ and can also quench azidyl radicals.^36^ It should be noted that blue light itself will photosensitise endogenous cellular chromophores, a process which could be affected by tryptophan, but the sensitivity of the cells themselves to blue light was insignificant (survival 103 ± 5.6%) and this was not significantly altered by the presence of tryptophan.

The irradiation of both **1** and **4** in the presence of 5′-GMP (2 mol eq.) in PBS produced the *mono*-5′-GMP Pt(ii) adduct *trans*-[Pt(py)_2_(5′-GMP)(N_3_)] (Fig. S9†). However, 23% of 5′-GMP was bound to platinum in the case of **1**, whereas only 9% was bound in the case of compound **4**, as judged by analysis of the starting/product 5′-GMP resonance S_1′_/P_1′_. This result, along with the UV-Vis and EPR spectroscopy studies demonstrates the enhanced photoreactivity of the carboxylate complex **1***versus* the dihydroxido complex **4**. TD-DFT calculations gave an insight into the enhanced photoactivity, showing that **1** has dissociative transitions which can extend up to 469 nm. The photoproducts from the irradiation of **1** with blue light (463 nm) were characterised by ^1^H NMR and ^195^Pt-NMR spectroscopy as well as LCMS. The release of succinate as well as azidyl radicals was observed (Fig. S10†). The reduction of Pt(iv) to Pt(ii) requires the gain of two electrons, however, the only radical detected by EPR spectroscopy was the azidyl radical. Since the major product generated after irradiation is a mono-azido Pt(ii) complex, the loss of both azides as azidyl radicals cannot be the main photochemical pathway to products in PBS. The second electron could originate from either the hydroxido or the carboxylato ligands, which could react rapidly with another radical or solvent molecule and thus be quenched.

The assignment of *trans*-[Pt(py)_2_(5′-GMP)(N_3_)] was readily confirmed as the ^1^H and ^195^Pt-NMR spectral data match those chemical shifts assigned previously for *trans*-[Pt(py)_2_(5′-GMP)(N_3_)].^29^ Irradiation of **1** with slightly shorter wavelength light (420 nm *vs.* 463 nm) did not produce in any significant change in the photoproducts, the change in irradiation wavelength appeared to only affect the rate of formation of products. Similar to the results obtained upon excitation at 463 nm, apart from the major product *trans*-[Pt(py)_2_(5′-GMP)(N_3_)], only very low intensity signals corresponding to other 5′-GMP adducts were visible in the ^1^H-NMR spectra.

LC-MS analysis was carried out after the irradiation of compound **1** in the presence of 2 mol eq. of 5′-GMP, with the majority of the species successfully assigned (Tables S1 and S6†). In H_2_O, in addition to *trans*-[Pt(py)_2_(5′-GMP)(N_3_)], other 5′-GMP adducts were observed including *trans*-[Pt(ACN)(GMP)(py)_2_]^2+^*trans*-[Pt(ACN)(GMP-H)(py)_2_]^+^, [Pt(N_3_)(py)_3_]^+^ and the di-platinum species [Pt_2_(μ-5′-GMP-H)(N_3_)_2_(py)_4_]^+^. The ACN adducts form due to use of acetonitrile as HPLC solvent, but in biological media, it is anticipated this coordination site would be readily occupied by another ligand. Irradiation in PBS (139 mM chloride) resulted in a chloride readily binding to give [Pt^II^(Cl)(py)_2_(5′-GMP)]^+^. This is consistent with a report that the amount of platinated DNA was significantly reduced when **4** was irradiated in the presence of 100 mM chloride.^51^ The [M − N_3_]^+^ species observed for **1** in the mass spectrum before irradiation (529.0750 *m*/*z*, Fig. S1,† top) was also observed post-irradiation, but only at low intensity (529.0790 *m*/*z*, peak 8, Fig. 6 and Table S6†). We suggest that in-source fragmentation and loss of azide may occur in the mass spectrometer in the absence of irradiation, but this does not seem to be significantly enhanced by irradiation. The corresponding [M − N_3_]^+^ species was also detected for **3**, before (562.1210 *m*/*z*, Fig. S1,† bottom) and following irradiation (562.1171 *m*/*z*, peak 8, Fig. 7 and Table S7†). This could be a Pt(iii) complex or a metallacyclic species in which the *N*-methylanthranilate ligand is doubly deprotonated and coordinates to Pt(iv) in a bidentate chelating mode. Pt(iii) intermediates are not unusual in mass spectrometry, but the elution of the species from the HPLC column suggests that it is stable, and therefore may be more likely to correspond to a metallacyclic Pt(iv) complex. Very little 5′-GMP binding was observed for **3** in 75% MeOH-*d*_4_/25% D_2_O (v/v) following irradiation, and photoproducts of **3** detected by LC-MS included the axial ligand (*N*-MI) (Fig. 7 and Table S7†), whereas for **1**, no assignable photoproducts retained the succinate ligand. In general, the photoproducts detected by LCMS were consistent with those previously seen following photoplatination of the synthetic oligodeoxynucleotide 5′dCATGGCT (containing two consecutive guanine nucleobases) by **1**.^15^

The attachment of fluorescent tags to platinum compounds is useful to study their temporal and spatial distribution in living cells. Frequently, the attachment of fluorescent tags to Pt(iv) results in fluorescence quenching, when reduced to Pt(ii), within the cell, fluorescence enhancement occurs.^52,53^ The fluorescence emission spectrum of **3** – which incorporates a fluorescent ligand (*N*-MI) in the axial position – showed a single broad band with maximum at 420 nm, similar to the maximum emission wavelength of the free ligand *N*-MIA, and repeated excitation at 320 nm caused an enhancement in the fluorescence emission at 420 nm, which is consistent with the detection of free *N*-MIA ligand by LCMS (Fig. S12†).

Pyridine release was not observed when complex **4** was irradiated with UVA light in aqueous solution.^29^ However, irradiation of either **3** or **4** in 75% MeOH-*d*_4_/25% D_2_O (v/v), released of pyridine, particularly upon UVA irradiation (Fig. S11†). This highlights the importance of the solvent and the environment on the photodecomposition pathways of these complexes; if these drugs accumulate in a hydrophobic, non-aqueous environment within the cell (*e.g.* membranes) different photoproducts may be produced. The release of pyridine is consistent with TD-DFT calculations performed on compounds **1** and **4** ^29^ which showed that the LUMO and LUMO+1 are strongly antibonding towards all ligands.

Complexes **1** and **2** showed promising photocytotoxicity towards ovarian cancer cells (A2780) with low-dose blue light (420 nm, 5 J cm^−2^). The phototoxicity of **1** and **2** are 2-fold higher compared to compound **4**. Complex **3**, was the least phototoxic with an IC_50_ value of 13.4 μM. This is despite complex **3** showing *ca.* 10-fold greater uptake in comparison to the rest of the compounds (Fig. 12, top). The comparatively modest IC_50_ for **3** may be related to the low yield of azidyl radicals or other factors, such as sub-cellular distribution. Cell uptake of **2** is *ca.* 2-fold higher than that of **1** in A2780 cells (from 15 to 25 ng/10^6^), attributable to the increased hydrophobicity arising from the propyl ester functionality in **2**. However, only a slight improvement in the cytotoxicity was observed (IC_50_ values of 2.7 μM for **1** and 3.7 μM for **2**). Complex **4** showed greater accumulation than both **1** and **2**, but lower photocytotoxicity, underlining the importance of triggered reduction and production of azido radicals in the biological effects.

The transport of molecules across the membrane of a cell can be either active (energy-dependent) or passive (energy-independent).^54^ Platinum(ii) complexes are taken up by both passive and active transport. For cisplatin, copper transporters (*e.g.* Ctr1) play a major role.^55–57^ Organic cation transporters constitute another class of important transporters, responsible for the uptake of picoplatin in mice.^58^ Cisplatin shows minimal cellular uptake at 4 °C (0.005 ng of Pt per 10^6^ cells) consistent with predominantly energy-dependent transport.^59^ In contrast, complexes **3** and **4** accumulated to a greater extent at this temperature, with accumulation increasing with temperature (Fig. 12, bottom). The uptake of these complexes is therefore likely to be through a combination of both passive and active transport, the passive transport appears to be enhanced greatly by the incorporation of an aromatic ligand in the axial position, as shown by complex **3**.

Complexes **1** and **2** were also more effective against the Pt-resistant A2780cis cell line compared to complex **4**. These three complexes all showed activity against OE19 oesophageal carcinoma cells with blue light (420 nm). Blue light is already in widespread clinical use for treating actinic keratoses – precancerous lesions on the skin, and has been shown to be more appropriate for treating superficial oesophageal cancerous lesions in animal models than green light, since it causes less damage to the underlying healthy tissue.^60^ For other applications however, longer-wavelength light activation is more desirable, and therefore the response of **1** and **2** to irradiation with green (517 nm) light and photocytotoxic activity towards both in ovarian cancer (and cisplatin-resistant ovarian cancer) cell lines is encouraging. The photocytotoxicities (*λ*_max_ 420 nm) of **1–3** are broadly consistent with guanidinoneomycin-derivatised succinate complexes in SK-MEL-28 cancer cell lines; in the SK-MEL-28 line the succinate complex **1** was less active than complex **4**,^15^ whereas complex **1** was slightly more active than **4** in the A2780, A2780cis and OE19 cell lines reported here.

## Conclusions

Complexes *trans,trans,trans*-[Pt(N_3_)_2_(OH)(OCOR)(py)_2_] where OCOR = succinate, 4-oxo-4-propoxybutanoate and *N*-methylisatoate (**1**, **2** and **3**, respectively), were synthesized and characterised by NMR spectroscopy, MS, UV-vis spectrometry and X-ray crystallography. Photoactivation studies comparing complexes **1–3** to their dihydroxido synthetic precursor *trans,trans,trans*-[Pt(N_3_)_2_(OH)_2_(py)_2_] **4** indicate a 3-fold faster rate of photodegradation for **1–3** through monitoring loss of the LMCT band in the UV-vis absorbance spectrum when irradiated with green light (517 nm). This is consistent with TD-DFT transitions indicating greater absorption at longer wavelengths for **1** in comparison to **4**. Consistent with this, complex **1** also showed significantly greater (2.6-fold) 5′-GMP complexation than **4** when irradiated with the same dose of blue (463 nm) light. EPR spectroscopic studies of **1**, **2** and **4** demonstrated that considerably higher quantities of azidyl radicals were generated under green light (517 nm) irradiation of **1** and **2**, with correspondingly lower (DMF/H_2_O) or minimal (PBS) radical production for **4**. Complex **3** incorporates the fluorescent ligand *N*-methylisatoate (*N*-MIA). Irradiation of **3** also resulted in an increase in fluorescence, consistent with platinum(iv) reduction and *N*-MIA release. LC-MS studies upon blue light irradiation (420 nm) resulted in the release of the axial ligands (succinate for **1** and *N*-MIA for **3**) and formation of a variety of photoproducts. Irradiation of **3** and **4** in non-aqueous solvent (75% MeOH-*d*_4_/25% D_2_O) in the presence of 5′-GMP also resulted in release of pyridyl ligand(s) from the complexes, which is not observed for **4** in aqueous or buffered solutions. The p*K*_a_ of the pendant carboxylate of complex **1** (5.13), suggests it may be negatively charged under biological conditions, which could explain the lower cellular uptake of **1** in comparison to **2** and **4**. Although complex **3** showed high levels of cellular accumulation, it showed poor aqueous solubility in comparison to **1** and **2**, and also lower azido radical trapping upon illumination with green light, possibly as a result of quenching by the *N*-MIA ligand. Cellular uptake studies indicated that complexes **3** and **4** may enter cells through a combination of passive diffusion and active transport.

Phototoxicity studies with 420 nm (blue) irradiation on A2780 cells showed that complexes **1** and **2** (IC_50_ = 2.7 and 3.7 μM) were ∼2-fold more photocytotoxic than **4** (IC_50_ = 5.1 μM). These complexes were also more effective towards the platinum-resistant A2780cis cell line in comparison to **4**, particularly complex **2**. The propyl derivative **2** was synthesised to produce a neutral compound, potentially also with greater lipophilicity than **1**, and although this demonstrated greater cellular accumulation in A2780 cells, this did not translate to greater photocytotoxicity than **1** in this cell line. Crucially, both complexes **1** and **2** also efficiently killed A2780 cells when activated by green (517 nm) light. Of the novel complexes, **3** was the least photocytotoxic (13.4 μM). Although the photoproducts formed by complexes **1**, **2** and **4** are similar, the improved photoactivity that the carboxylate ligand confers to **1** and **2** results in rapid generation of Pt(ii) photoproducts and higher concentrations of azide radicals for a given irradiation dose.

The derivatisation of one axial position of platinum(iv) azido complexes with carboxylate ligands enables them to be activated with green (517 nm) light, whilst remaining stable in the presence of biological reductants (GSH). Complexes **1** and **2** have the potential to be further modified or developed in their own right as phototherapeutics, particularly for superficial cancers; complex **2** in particular showed promising activity towards the cisplatin-resistant A2780cis cell line. The production of azido radicals by the complexes is clearly of crucial importance to their photocytotoxic mechanism of action.

## Experimental

Synthesis and characterisation of synthetic precursors **5**, **6** and **7** and **4** are given in the ESI.† The NMR spectroscopic assignment of specific hydrogens and carbons for complexes **1–3** is illustrated in Fig. S13.†

***Caution!*** No problems were encountered during this work, however heavy metal azides are known to be shock-sensitive detonators, therefore it is essential that platinum azides are handled with care. The Pt-diazido complexes were synthesised and handled under dim lighting conditions.

### *trans,trans,trans*-[Pt(N_3_)_2_(OH)(succ)(py)_2_](**1**)

Carboxylation of one of the axial ligands of **4** was achieved by modifying a published method.^61^*trans,trans,trans*-[Pt(N_3_)_2_(py)_2_(OH)_2_] **3** (0.150 g, 0.318 mmol) was dissolved in anhydrous DMSO (1.8 mL) under N_2_, in the presence of activated molecular sieves. Succinic anhydride (1.1 mol eq., 0.035 g) was added and the reaction stirred in the dark at 40 °C for 48 h. The suspension was filtered and water (∼2 mL) was added. The yellow solution was allowed to stand at room temperature until a crystalline solid precipitated (3–4 h). The remaining solution was decanted and the crystalline precipitate was washed with diethyl ether and then filtered (yield = 0.096 g, 52%). Crystals suitable for single-crystal X-ray diffraction were obtained by slow diffusion of water into the DMSO reaction mixture at room temperature. Purity (HPLC, UV-vis absorbance): 94%.

^1^H-NMR (D_2_O, 600 MHz, pH 5.18, ppm): *δ* = 8.77 (dd, ^3^*J*_^1^H^1^H_ = 5.78 Hz, ^3^*J*_^195^Pt^1^H_ = 11 Hz, 4H, H_o_), *δ* = 8.27 (t, ^3^*J*_^1^H^1^H_ = 7.64 Hz, 2H, H_p_), *δ* = 7.80 (t, ^3^*J*_^1^H^1^H_ = 7.04 Hz, 4H, H_m_,), *δ* = 2.58 (t, ^3^*J*_^1^H^1^H_ = 6.90 Hz, 2H, H_1_), *δ* = 2.48 (t, ^3^*J*_^1^H^1^H_ = 6.90 Hz, 2H, H_2_). ^13^C-NMR (*d*_4_-MeOD, 150 MHz, ppm): *δ* = 178.7, (C_a_), *δ* = 177.57 (C_d_), *δ* = 150.89, (C_e_), *δ* = 143.15 (C_g_), *δ* = 127.37 (C_f_). ^195^Pt-NMR (D_2_O, 129 MHz): *δ* = 1059 ppm.

ESI-MS: [M + Na]^+^ (*m*/*z*) calc. (C_14_H_16_N_8_NaO_5_Pt), 594.0741; found, 594.0783.

### *trans,trans,trans*-[Pt(N_3_)_2_(OH)(succ-Pr)(py)_2_](**2**)

*trans,trans,trans*-[Pt(N_3_)_2_(OH)(Succ-Pr)(py)_2_] **4** (0.100 g, 0.176 mmol) was pre-dried using P_2_O_5_ and then dissolved in dry DMF (1 mL) under argon. A stock solution of CDI (1,1′-carbonyldiimidazole) in DMF (0.194 M) was prepared under dry conditions and 1.2 mL was added (1.2 mol eq.) to the solution of the platinum complex and allowed to stir at 50 °C for 20 min, under continuous purging with argon. Then, sodium propanolate (2 mL), formed by the dissolution of sodium metal (0.02 g) in absolute propanol (20 mL), was added and the reaction carried out at room temperature for 48 h. DMF was removed under vacuum and the crude product was purified by column chromatography (eluent: EtOAc : MeOH, 11 : 1). After solvent removal from the combined fractions, the solid was recrystallised from DCM/diethyl ether (yield = 0.060 g, 56%). Crystals suitable for X-ray crystallography were grown by slow evaporation from H_2_O at ambient temperature. Purity (HPLC, UV-Vis absorbance): 96%.

^1^H-NMR (D_2_O, 600 MHz, dioxane, ppm): *δ* = 8.76 (dd, ^3^*J*_^1^H^1^H_ = 5.64 Hz, ^3^*J*_^195^Pt^1^H_ = 25.40 Hz, 4H, H_6_), *δ* = 8.28 (t, ^3^*J*_^1^H^1^H_ = 7.73 Hz, 2H, H_7_), *δ* = 7.81 (t, ^3^*J*_^1^H^1^H_ = 7.14 Hz, 4H, H_8_), *δ* = 3.95 (t, ^3^*J*_^1^H^1^H_ = 6.72, 1H, H_3_), *δ* = 2.64, 2.63 (m, 2H, H_1,2_), *δ* = 1.54 (s, ^3^*J*_^1^H^1^H_ = 7.28 Hz, 1H, H_4_), *δ* = 0.84 (t, ^3^*J*_^1^H^1^H_ = 7.50 Hz, 1H, H_5_). ^13^C-NMR (D_2_O, 150 MHz, dioxane, ppm): *δ* = 179.29 (C_d_), *δ* = 176.22 (C_a_), *δ* = 149.84 (C_h_), *δ* = 143.31 (C_j_), *δ* = 127.64 (C_i_), *δ* = 67.90 (C_e_), *δ* = 31.78 (C_b_), *δ* = 30.88 (C_c_), *δ* = 21.91 (C_f_), *δ* = 10.17 (C_g_). ^195^Pt-NMR (D_2_O, 107 MHz, dioxane): 1065 ppm. ESI-MS: [M + Na]^+^ (*m*/*z*) calc. (C_17_H_22_N_8_NaO_5_Pt). 636.1210; found, 636.2.

### *trans,trans,trans*-[Pt(N_3_)_2_(OH)(*N*-MI)(py)_2_](**3**)

*trans,trans,trans*-[Pt(N_3_)_2_(py)_2_(OH)_2_] **3** (0.086 g, 18.25 mmol) was dissolved in anhydrous DMSO (2 mL) and *N*-methylisatoic anhydride (0.162 g, 91.23 mmol, 5 mol eq.) was added. The reaction was stirred for 72 h at 40 °C under nitrogen with an outlet to allow the release of CO_2(g)_. The isolation of the product was achieved by the addition of water (10 mL) at which point the compound precipitated. For the removal of excess ligand, repeated washing with diethyl ether was necessary and carried out *via* centrifugation (yield = 0.020 g, 18%). Crystals suitable for X-ray diffraction were grown by slow evaporation of a solution in DCM/MeOH at ambient temperature. Purity (HPLC, UV-vis absorbance): 94%.

^1^H-NMR (DMSO-d_6_, 500 MHz, ppm): *δ* = 8.86 (dd, ^3^*J*_^1^H^1^H_ = 5.54 Hz, ^3^*J*_^195^Pt^1^H_ = 27.00 Hz, 4H, H_7_), *δ* = 8.30 (t, ^3^*J*_^1^H^1^H_ = 7.56 Hz, 2H, H_9_), *δ* = 7.88 (m, 5H, 4H_8_, H_4_), *δ* = 7.62 (q, ^3^*J*_^1^H^1^H_ = 5.07 Hz, 1H, H_5_), *δ* = 7.26 (t, ^3^*J*_^1^H^1^H_ = 7.78 Hz, 1H, H_3_), *δ* = 6.53 (d, ^3^*J*_^1^H^1^H_ = 7.53 Hz, 1H, H_1_), *δ* = 6.51 (t, ^3^*J*_^1^H^1^H_ = 7.53 Hz, 1H, H_2_), *δ* = 3.88 (d, ^2^*J*_^195^Pt^1^H_ = 14.8 Hz, H_10_), *δ* = 2.70 (d, ^3^*J*_^1^H^1^H_ = 5.00 Hz, 3H, H_6_). ^13^C-NMR (DMSO-*d*_6_, 125 MHz, ppm): *δ* = 171.26 C_a_, *δ* = 150.82 C_c_, *δ* = 149.73 C_b_, *δ* = 149.38 C_i_, *δ* = 142.18 C_k_, *δ* = 132.86 C_g_, *δ* = 132.09 C_f_, *δ* = 126.42 C_j_, *δ* = 113.74 C_d_, 110.00 C_e_, *δ* = 29.07 C_h_. ^195^Pt-NMR (DMSO-*d*_6_, 107 MHz,): *δ* = 990 ppm. ESI-MS: [M + H]^+^ (*m*/*z*) calc. (C_18_H_20_N_9_O_3_Pt) 605.1288; found, 605.1338.

## Conflicts of interest

There are no conflicts to declare.

## Supplementary Material

DT-050-D1DT01730F-s001

DT-050-D1DT01730F-s002
