# Supplementary material for: Platinum(iv)-azido monocarboxylato complexes are photocytotoxic under irradiation with visible light
Source: Dalton Trans. 2021 Jul 12;50(30):10593–607. doi: 10.1039/d1dt01730f (PMC8335519; doi:10.1039/d1dt01730f)
Supplement: DT-050-D1DT01730F-s001 [file DT-050-D1DT01730F-s001.pdf]

Electronic Supplementary Material (ESI) for Dalton Transactions.
This journal is © The Royal Society of Chemistry 2021

Electronic Supporting Information

Platinum(IV)-azido monocarboxylato complexes are photocytotoxic under irradiation with visible light

Evyenia Shaili, María J. Romero, Luca Salassa, Julie A. Woods, Jenny S. Butler, Isolda Romero-Canelón, Guy J. Clarkson, Abraha Habtemariam , Peter J. Sadler, Nicola J. Farrer

Materials and Methods

Tables S1-S7

Figures S1-S14

References

Materials and Methods

Materials: $K_2[PtCl_4]$ was purchased from Precious Metals Online. HPLC-grade solvents and Millipore filtered H_2O were used for the preparation and purification of compounds by HPLC. All other reagents and consumables were purchased from commercial suppliers and used as received. (IM) indicates use of a nylon syringe filter (pore size 0.2 μM). All manipulations were carried out under reduced lighting and solutions were prepared stored and handled with minimal exposure to light.

NMR spectroscopy: ^1H, ^{13}C and ^{195}Pt NMR spectra were acquired at 298 K on Bruker AV-400 (^1H: 399.10 MHz), Bruker DPX-400 (^1H: 400.03 MHz) or Bruker AVIII-600 (^1H: 600.13 MHz) spectrometers. Samples were prepared by the addition of the appropriate solvent (0.6 mL) in 5 mm NMR tubes. 1D ^1H-NMR spectra were recorded using standard pulse sequences. Typically the data were acquired with 128 or 16 transients into 32 k data points over a spectral width of 14 ppm. The ^1H-NMR chemical shifts were internally calibrated with the residual peaks of solvent CHD_2COCD_3 (δ = 2.05 ppm), CHD_2OD (δ = 3.31 ppm), DMSO-d_6 (δ = 2.50 ppm) or added 1,4-dioxane (δ = 3.75 ppm) in the case of D_2O.[1] All the novel Pt(IV) complexes were characterized by ^{13}C-NMR spectroscopy. The pulse sequences used were jmod (J Modulation) and PENDANT (Polarization ENhancement During Attached Nucleus Testing). In both cases, the peaks for $–CH_2$ or quaternary carbons appear to be inverted relative to those for –CH or CH_3 groups. The signals of quaternary carbons were generally of low intensity.[2]
COSY (Correlation spectroscopy) spectra were recorded to identify pairs of nuclei which are coupled through bonds. Typically they were acquired using 8 transients into 1024 data points. [^1H, ^{13}C] HSQC (Heteronuclear Single-Quantum Correlation) 2D spectra were used for the assignment of ^{13}C NMR peaks. The spectra were acquired with 32 transients and the time domain was 1024 or 2048.
The ^{195}Pt-NMR spectra were typically obtained with pulse sequence zg (non-proton decoupled), with a relaxation delay (D1) of 0 s, using a 2 k or 65 k time domain and 20 k transients. The spectra were externally referenced with K_2PtCl_6 (15 mM in D_2O).
Water suppression was used when samples were prepared in aqueous solution. To minimise the large residual water peak, the HOD signal was suppressed by irradiating at the frequency of water in between pulse sequences.

Electrospray mass spectroscopy (ESI-MS): Positive-ion ESI spectra were obtained by preparing the samples in CH_3OH or H_2O and infusing into the mass spectrometer (Bruker Esquire 2000 Trap Spectrometer or Agilent 6130 single Quad), using an automatic sample delivery system. The mass spectra were recorded with a scan range of *m/z* 500-1000 for positive ions. Data were processed using Data Analysis version 3.3 (Bruker Daltonics). The detection of platinum-containing species was aided by the distinctive isotopic distribution pattern of platinum.

X-ray crystallography: Diffraction data were collected by the use of MoKα radiation at a wavelength of λ=0.71073 Å on an Xcalibur Gemini diffractometer with Ruby CCD area detector. The crystals were held at the following temperatures: 296 K (complex **1**) 150 K

(complexes **2** and **3**) during data collection. Using Olex2,[3] the structures were solved with the XS[4] structure solution program using Direct Methods and refined with the ShelXL[5] refinement package using Least Squares minimisation. The OH and NH hydrogen atoms were located in a difference map but refined with restraints. Images representing the intermolecular forces for each complex were created using Mercury 3.3.
X-ray crystallographic data for complexes **1–3** have been deposited in the Cambridge Crystallographic Data Centre under the accession numbers 1998067, 1998068 and 1998069 respectively. X-ray crystallographic data in CIF format are available from the Cambridge Crystallographic Data Centre (http://www.ccdc.cam.ac.uk/).

UV-vis absorption spectra: were recorded on a Varian Cary 300 UV-vis spectrophotometer in 1 cm path-length quartz glass cuvettes, purchased from Starna Scientific. The spectral width was 200 – 800 nm, with bandwidth 1.0 nm and a scan rate of 600 $nm.min^{-1}$.

Fluorescence measurements: The emission spectra were recorded on a Jasco FP-6500 using the following parameters: excitation wavelength at 320 nm, excitation and emission slits at 10 nm and 5 nm respectively. The response was set to medium and the scanning rate to 200 nm. min^{-1}.

pKa determination of complex 1: pH values were measured in NMR tubes with a solid-state electrode pocket ISFET pH meter (Minilab Series). The meter was calibrated using standard buffer solutions (pH 7.00, pH 4.01, pH 10.01, Minilab). Samples were dissolved in D_2O (1.1 mM) and the pH* was adjusted to the desired value by using 0.01 M, 0.05 M, 0.1 M KOD and 0.1 M, 0.05 M, 0.001 M $DClO_4$ solutions. Values determined in D_2O were converted to values for H_2O according to the equation $pK^{H} = 0.929\ pK^{H*} + 0.42$.[6]

Exponential rate fitting: For determining the rate of loss of LMCT bands in UV-vis spectra, the maximum for each of the graphs was normalized so that the first spectrum (t=0 h, i.e. before the irradiation) had a normalized absorbance of 100%. Then by considering only the initial degradation (up to *ca.* 38%) the points were fitted to an exponential curve ($y=y_0+Ae^{Rox}$) using Origin 8.5.

Electron Paramagnetic Resonance: The EPR spectra were recorded on a Bruker EMX (X-band) spectrometer. A cylindrical T_{M10} mode cavity (Bruker 4103 TM) was used and samples were placed in spectrosil quartz tubes of inner diamater 1.0 mm and outer diameter of 1.2 mm (Wilmad Labglass) sealed with J-Blue Tac. The tube was placed inside a larger quartz tube so the sample could be more accurately positioned inside the resonator. Parameters: modulation amplitude 2.0 G, microwave power 0.63 mW, 1.0 $x10^4$ receiver gain, sweep gain at 41.94 s and a repeated number of 10 X scans was used and resolution in Y of 24. The green LED (517 nm) was placed at a distance of 8.5 cm from the tube in the EPR cavity. Irradiations lasted for 35 min; every 7 min a spectrum was recorded. The spin adduct concentration was determined using a calibration curve (SigmaPlot) obtained from standard solutions of 4-hydroxy-2,2,6,6-tetramethyl-piperidine-1-oxyl (Tempol) in the corresponding solvent and the processing was carried out using Bruker WINEPR software.

Computational details: All calculations were performed with the Gaussian 03 (G03) program[7] employing the DFT method, the PBE1PBE[8] functionals. The LanL2DZ basis set[9] and effective core potential were used for the Pt atom and the 6-31G**+ basis set[10] was used for all other atoms. Geometry optimizations of complex **1** (deprotonated) in the ground state (S_0) and lowest-lying triplet state (T_1) were performed in the gas phase and the nature of all stationary points was confirmed by normal mode analysis. For the T_1 geometry the UKS method with the unrestricted PBE1PBE functional was employed. The conductor-like polarizable continuum model method (CPCM)[11] with water as solvent was used to calculate the electronic structure and the singlet excited states of complex **1** in solution (H_2O). Thirty-two singlet excited states with the corresponding oscillator strengths were determined with a Time-dependent Density Functional Theory (TD-DFT)[12,13] calculation.

LC-MS studies: LC-MS analysis was carried with a Dionex 3000RS UHPLC coupled with Bruker MaXis Q-TOF mass spectrometer. An Agilent Zorbax Eclipse plus column (C18, 100x2.1 mm, 1.8 μm) was used. Mobile phases were A (water with 0.1% formic acid) and B (as ACN with 0.1% formic acid). A gradient of 5% B to 100% B in 15 min was employed with flow rate at 0.2 $mL.min^{-1}$, UV was set at 240 nm. The mass spectrometer was operated in electrospray positive mode with a scan range 50-2,000 *m/z*. Source conditions: end plate offset at -500 V; capillary at -4500 V; nebulizer gas (N_2) at 1.6 bar; dry gas (N_2) at 8 $L.min^{-1}$; dry temperature at 180 °C. Ion transfer conditions: ion funnel RF at 200 Vpp; multiple RF at 200 Vpp; quadruple low mass set at 55 *m/z*; collision energy at 5.0 eV; collision RF at 600 Vpp; ion cooler RF at 50-350 Vpp; transfer time set at 121 μs; pre-Pulse storage time set at 1 μs. Calibrations employed sodium formate (10 mM) injected through a loop injection of 20 μL of standard solution at beginning of each run.

Irradiation methods and devices: The light sources used for photoactivation were as follows: (a) LZC-ICH2 photoreactor (Luzchem Research Inc.) equipped with a temperature controller and 3 UVA lamps (Hitachi, λ_{max} = 361 nm) or 6 Luzchem LZC-420 lamps (λ_{max} = 420 nm) with no other sources of light filtration; (b) LED light sources (BASETech model no. SP-GU10 230V~50 Hz 1.3-2.1 W) with λ_{max} = 463 nm or 517 nm; (c) KiloArc™ broadband arc lamp monochromator, which was supplied with the appropriate filters to remove shorter wavelength light. The light intensity used to irradiate samples was measured with an International Light Technologies Powermeter (ILT1400-A) equipped with SEL 033 detector and either a UVA/TD filter (315-390 nm) for UVA irradiations or a flat response visible filter F/W (400-1064 nm) for visible wavelengths. The determination of the light dose in the experiments was calculated by the equation: Dose ($mJ\ cm^{-2}$) = Time (s) × Irradiance ($mW\ cm^{-2}$).

Phototoxicity testing: Cell culture and other chemicals were obtained from Sigma Aldrich Ltd (Poole, UK). Disposable sterile cell culture plastics were obtained from Greiner Bio-One (Cambridge, UK). All procedures were carried out in a specially adapted photobiology laboratory with ambient light levels measured below 1 lux (Solatell, UK).

OE19 human oesophageal carcinoma and the paired A2780/A2780cis human ovarian carcinoma cell lines were obtained from the European Collection of Cell Cultures (Porton Down, UK) and maintained in RPMI containing 10% (v/v) foetal calf serum. Cells were free

of mycoplasma and were maintained in antibiotic-free conditions in a humidified atmosphere of 5% CO_2/95% air. For experiments, cells were seeded at a density of 6 -7 x 10^4 cells.cm^{-2} in 96 well plates for broadband irradiation; or 2 x 10^6 cells.mL^{-1} in stirred culture for monochromatic irradiation. Complexes were prepared immediately before use in Earle's Balanced Salt Solution (EBSS) and filter-sterilised, with the exception of complex **3** which was dissolved in DMSO and then diluted into EBSS. Irradiations were performed in optically clear medium and experiments were controlled for light, complex, solvent (when required), and handling. Blue light was delivered by a bank of TL03 fluorescent tubes (λ_{max}: 420 nm) with wavelengths shorter than 400 nm blocked by filtering. Irradiance was measured with a Gigahertz Optik meter calibrated to the source using a spectroradiometer (Bentham, UK; mean irradiance 1.3 mW.cm^{-2} ± 0.3). Irradiances were measured through filters, and where appropriate, cell culture plate lids. Monochromatic irradiation was performed on stirred cultures using a Bentham monochromator equipped with a 450 W Xenon arc lamp and light guide. Irradiance was measured using a calibrated integrating sphere and all measurements were traceable to the National Physics Laboratory (mean irradiance at 517 ± 27 nm: 97.5 mW.cm^{-2} ± 3.8; at 570 ± 27 nm: 49.0 mW.cm^{-2} ± 1.7). Sham-irradiated cells were treated identically and in parallel with irradiated cells, except that photons were blocked.

Phototoxicity was determined by neutral red dye uptake either 24 hours after irradiation (broadband irradiation) or alternatively cells were seeded at a low density and toxicity measured 72 hours post-irradiation (monochromatic irradiation). Absorbance was read at 540 nm in a Synergy™ 2 plate reader. The concentration of complex required to inhibit dye uptake by 50% (IC_{50} value) was calculated from the log-transformed cytotoxicity curves normalised to untreated cells (Graphpad Prism v.6). Goodness of fit was determined by the 95% confidence interval of the IC_{50} value, and the R^2 value. The phototoxic index (PI) indicates the fold difference in cytotoxicity between cells treated with complex and either sham-irradiated or irradiated. Cells were also seeded at a low density into 35 cm dishes and stained with crystal violet (0.5%) 7-10 days post-irradiation. Experiments were performed in triplicate, and independently repeated at least once (6 observations from n=2) on cells of differing passage number.

The comet assay was performed as previously described.[14] In order to reveal DNA adducts that inhibit DNA migration, the cells are briefly incubated (5 mins) with a low dose of hydrogen peroxide (25 μM) after the cells have been photoactivated and are about to be incorporated into the agarose gel and lysed in a DMSO-containing solution. To show DNA lesions that increase DNA migration (i.e. strand breaks), no peroxide is added. After electrophoresis, the nuclei were stained with ethidium bromide and scored by eye using a Nikon E600 eclipse fluorescent microscope.

Cell uptake studies: A2780 cells were seeded at a density of $5x10^5$ cells.mL^{-1} in each well and they were allowed to adhere for 36 h. Before the complexes were added the cells were washed with PBS (1 ml). The stock concentration of the complex was confirmed via ICP-MS analysis before the complex exposure to the cells. The complexes (20 μM, 2 mL in PBS) were added and allowed to incubate for 1 h, in the dark at 37 ^oC. Following the uptake of the complex, cells were washed with PBS (1 mL) and a solution of Trypsin/ EDTA (2%) was added (0.5 mL) and

incubated at 37 °C for 3 min, and then neutralized with cell culture media-RPMI (2 mL). Cells were transferred into falcon tubes and broken into a single cell suspension to enable counting, additional RPMI (2 mL) was added to dilute the cells. Cells were counted in duplicate and an average was taken. They were then spun down (1000 rpm, 22 C°, 5 min). The supernatant was removed and the cells were resuspended in PBS (1 mL) for a further wash. The cell pellet was kept at -20 °C until the Pt content was analysed via ICP-MS.

ICP-MS: Digestion of the cell pellet was carried out overnight in concentrated nitric acid (73%) at 70 °C. The resulting solutions were diluted to 5% *v/v* HNO_3, using doubly ionised water and then filtered via an Inorganic Membrane filter to remove any insoluble cell debris. Platinum content was measured by ICP-MS.

Temperature-dependent cell uptake experiments: were performed for complexes **3** and **4**, at 4 °C, 18 °C and 37 °C, after 1 h complex exposure in the dark. Each experimental condition was carried out in triplicate.

HPLC purity test: The purity of the compounds was performed on Agilent 1200 system with a VWD and 100 µL injection loop. The column used was an Agilent ZORBAX Eclipse Plus C18, with dimensions of 250 x 4.6 mm and a 5 µm port size. The injected volume was 45 µL. The mobile phase used was H_2O 0.1 % TFA/ ACN 0.1% TFA. The flow rate was set to 1mL.min^{-1} and the following method (**Table S1**) was used.

Table S1: HPLC method for determining the analytical purity of the compounds. Solvent B is ACN (0.1% TFA) and solvent A is H_2O (0.1% TFA).

**Time (min)**	**%B**
0	10
30	80
40	80
41	10
55	10

Complexes **1** and **2** were dissolved in water and complex **3** in 10% MeOH at a concentration of *ca* 50 µM.

Synthesis and characterisation of synthetic precursors

***Caution!* No problems were encountered during this work, however heavy metal azides are known to be shock-sensitive detonators, therefore it is essential that platinum azides are handled with care. The Pt-diazido complexes were synthesised and handled under dim lighting conditions.**

N-Methylisatoic acid (N-MIA) (5)

The open form of the N-methylisatoic anhydride was prepared by hydrolysis and decarboxylation of N-methylisatoic anhydride.[15] N-MI anhydride (1.2 g, 6.77 mmol) was dissolved in a solution of 2 M KOH (15 mL) and allowed to react for 4 h at 100 ^{o}C. The transparent solution was then allowed to cool down at room temperature, before the pH was adjusted to 6 - 7 by the addition of HCl (3 M). The white precipitate which formed was isolated by filtration and dried under *vacuo* (Yield = 0.17 g, 19%). This compound was air-sensitive and was stored under nitrogen following preparation.

^{1}H-NMR (75% MeOH-d_4 / 25% D_2O, 400 MHz, ppm): δ= 8.45 (d, 8.29 Hz, H_1), δ= 7.39 (t, 7.74 Hz, H_2), δ= 6.78 (d, 8.21 Hz, H_3), δ= 6.66 (t, 7.57 Hz, H_4), δ= 2.87 (s, -CH_3). ESI-MS: $[M+Na]^+$ (*m/z*) Calc. 174.0; Found, 174.0, $[M+K]^+$ (*m/z*) Calc. 190.0; Found, 190.0.

***Trans*-[$PtCl_2(py)_2$] (6)**

K_2PtCl_4 (4 g, 9.64 mmol) was dissolved in H_2O (100 mL) and pyridine was added (15 mL, 193 mmol, 20 mol eq). The solution was stirred at 85 ^{o}C for 1.5 h after which point it became transparent. The solution was allowed to cool down and the solvent was removed by evaporation to yield a white solid which was washed with diethyl ether for the removal of excess pyridine. HCl (2 M, 100 mL) was added and the solution was heated at 75 ^{o}C overnight to give *trans*-[$PtCl_2(py)_2$] as a yellow solid which was filtered off and washed with ice-cold water, ethanol and diethyl ether (Yield = 3.75 g, 8.86 mmol, 92%).

^{1}H-NMR (acetone-d_6, 400 MHz, ppm): δ= 8.87 (dd, $^{3}J_{1H1H}$= 6.8 Hz, $^{3}J_{195Pt1H}$ = 31.1 Hz, 4H_o), δ= 8.02 (t, $^{3}J_{1H1H}$ = 7.7 Hz, 2H_p), δ= 7.52 (t, $^{3}J_{1H1H}$ = 7.5 Hz, 4H_m).

***Trans*-[$Pt(N_3)_2(py)_2$] (7)**

This complex was prepared as previously reported.[16] *Trans*-[$PtCl_2(py)_2$] (**1**) (3.75 g, 8.85 mmol) was suspended in water (500 mL), $AgNO_3$ (3.008 g, 2 mol eq) was added and the reaction was stirred at 60 ^{o}C overnight. The grey precipitate was filtered through celite over a frit to obtain a pale yellow solution which was then further passed through an IM filter. Then NaN_3 (5.75 g, 10 mol eq) was added and the reaction was stirred overnight at room temperature. The yellow precipitate was filtered and washed with ice-cold water, ethanol and diethyl ether (3.48 g, 90%). The solid was recrystallised from pre-warmed pyridine at 40 ^{o}C at a ratio of 1 g/37 mL of pyridine and following hot filtration it was allowed to crystallise at -20°C. The yellow solid was filtered, washed and dried under vacuum (Yield = 2.44 g, 70%).

^{1}H-NMR (acetone-d_6, 400 MHz, ppm): δ = 8.86 (dd, $^{3}J_{1H1H}$ = 6.7 Hz, $^{3}J_{195Pt1H}$ = 37.3 Hz, 4H_o), δ = 8.13 (tt, $^{3}J_{1H1H}$ = 7.7 Hz, $^{4}J_{1H1H}$ = 1.4 Hz, 2H_p), δ = 7.67 (t, $^{3}J_{1H1H}$ = 7.2 Hz, 4H_m).

***Trans, trans, trans*-[Pt(N_3)$_2$(OH)$_2$(py)$_2$] (4)**

The title product was obtained by oxidation of *trans*-[Pt(py)$_2$(N_3)$_2$].[16] *Trans*-[Pt(N_3)$_2$(py)$_2$] (2.00 g, 4.56 mmol) was suspended in H_2O_2 (160 mL, 30%) and allowed to react at 45 ^{o}C for 4 h, producing a yellow solution. This was filtered (IM) and transferred to a larger flask to which H_2O (340 mL) was added. The solution was lyophilized, yielding a yellow solid which was recrystallised from 2:1 ethanol/methanol and the product precipitated by addition of diethyl ether at -20 ^{o}C. The solid was filtered and dried under vacuum (Yield = 1.6 g, 80%).

^{1}H-NMR (D_2O, 400 MHz, dioxane, ppm): δ= 8.80 (dd, $^{3}J_{1H1H}$ = 6.7 Hz, $^{3}J_{195Pt1H}$ = 26.9 Hz, 4H$_o$), δ= 8.28 (tt, $^{3}J_{1H1H}$ =7.8 Hz, $^{3}J_{1H1H}$ =1.3 Hz, 2H$_p$), δ= 7.82 (t, $^{3}J_{1H1H}$ = 7.1 Hz, 4H$_m$).

ESI-MS spectra

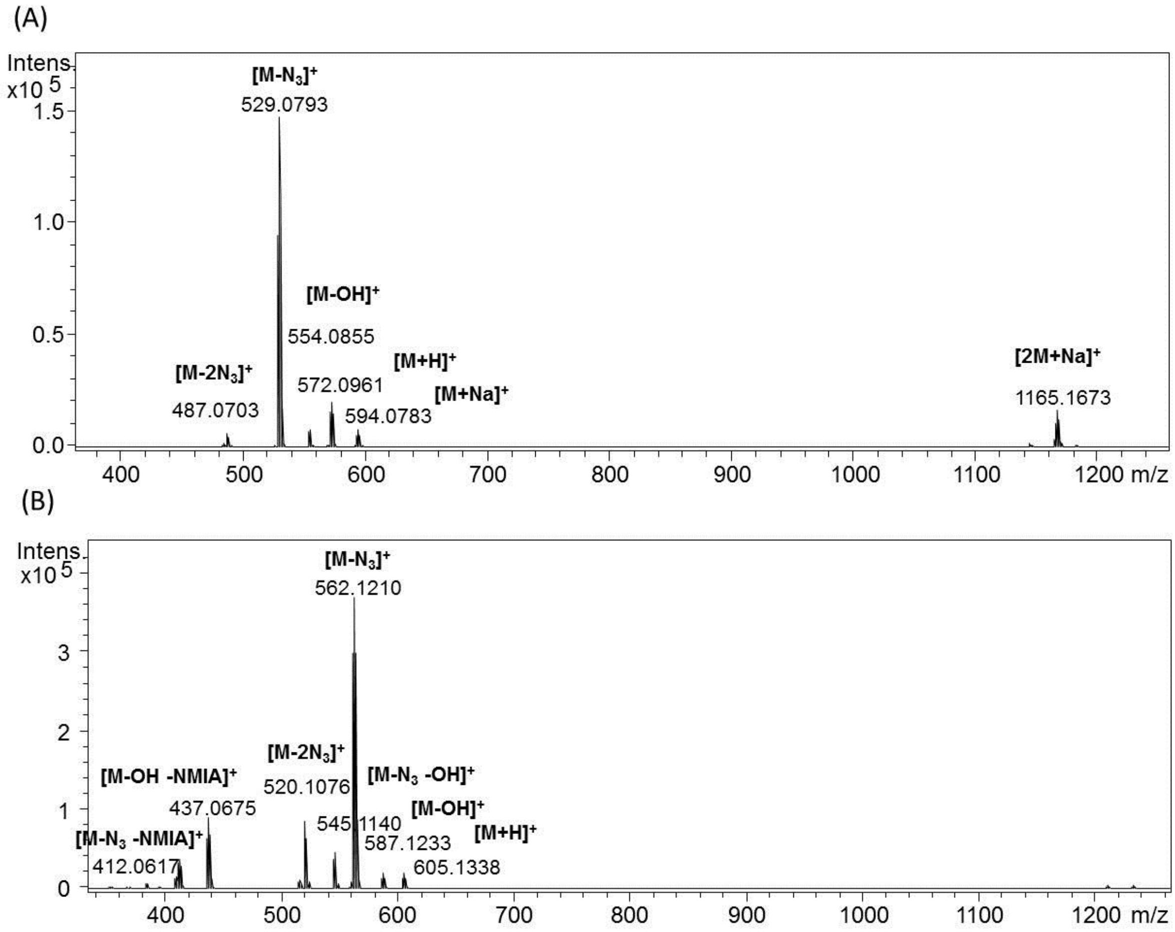

Figure S1: ESI-MS (H_2O/ACN, 0.1% TFA) of complex **1** (top) and **3** (bottom).

X-Ray crystallography

Table S2a: Crystallographic data for complexes **1**, **2** and **3**.

**Complex**	**1**	**2**	**3**
Empirical formula	$C_{14}H_{16}N_8O_5Pt$	$C_{17}H_{22}N_8O_5Pt$	$C_{18}H_{19}N_9O_3Pt$
Formula weight	571.44	613.52	604.51
Crystal system	Triclinic	Monoclinic	Monoclinic
Crystal size/ mm	0.40x0.10 x 0.10	0.40 x 0.08 x 0.08	0.30x 0.18 x 0.12
Space group	P-1	C2/c	P2(1)/c
a/ Å	8.4300(2)	27.4103(18)	7.5372(2)
b/ Å	10.5284(2)	12.5601(7)	35.8705(8)
c/ Å	11.9980(4)	13.6542(8)	8.1728(2)
α/(°)	86.002(2)	90	90
β/(°)	70.799(3)	115.8(9)	109.178(2)
γ/(°)	68.795(2)	90	90
Volume/ $Å^3$	936.07(4)	4252.4(4)	2086.99(9)
Z	2	8	4
D_{calc}/gcm^{-3}	2.027	1.917	1.924
F(000)	548	2384	1168
μ_{calcd} /mm^{-1}	7.540	6.646	6.764
T/ K	296(2)	150(2)	150(2)
2Θ range for data collection/°	7.204 to 61.296	5.642 to 63.008	5.398 to 57.998
Reflections collected/unique [R_{int}]	17109/5253 [0.0377]	14993/6342 [0.0385]	26941/5459 [0.0308]
Goodness-of-fit on F^2	1.070	0.997	1.409
Data/restraints/ parameters	5253/0/255	6342/0/282	5459/0/282
Final R indices [I>2σ(I)]	R_1= 0.0175, wR_2= 0.0408	R_1=0.0328, wR_2= 0.0480	R_1=0.0344, wR_2= 0.0535
Final R indices (all data)	R_1= 0.0189 wR_2= 0.0415	R_1= 0.0541 wR_2= 0.0553	R_1= 0.0392 wR_2= 0.0541
Largest diff. peak and hole (eÅ^{-3})	1.14 and -0.56	1.91 and -2.24	1.31 and -2.39

Table S2b: Selected bond distances (Å) and angles (°) for complexes **1**, **2**, **3** and the precursor complex **4**

Bond (Å)/ Angle (º)	1	2	3	4[a]
**Pt–O1**	2.0196(16)	2.056(2)	2.030(2)	1.990(3)
**Pt–O2**	1.9852(15)	1.971(2)	1.978(2)	2.027(3)
**Pt–N1**	2.040(2)	2.032(3)	2.025(3)	2.047(3)
**Pt–N2**	2.032(2)	2.038(3)	2.036(3)	2.047(3)
**Pt–N3**	2.049(2)	2.057(3)	2.053(3)	2.043(3)
**Pt–N6**	2.045(2)	2.052(3)	2.037(3)	2.047(3)
**N6–N7**	1.206(3)	1.216(4)	1.215(4)	1.215(4)
**N7–N8**	1.137(3)	1.151(4)	1.148(5)	1.139(4)
**N3–N4**	1.202(3)	1.210(4)	1.210(4)	1.218(5)
**N4–N5**	1.126(4)	1.150(5)	1.142(4)	1.146(5)
**N6–Pt–N3**	175.38(7)	177.44(13)	178.03(13)	176.86(12)
**N7–N6–Pt**	122.00(17)	115.9(2)	116.2(3)	118.0(3)
**N4–N3–Pt**	118.17(17)	115.7(2)	116.9(3)	120.3(2)
**N1–Pt–N2**	176.76(7)	178.45(12)	178.46(12)	179.09(12)
**O2–Pt-O1**	174.21(7)	175.52(9)	176.50(11)	179.58(11)
**N3–N4-N5**	174.3(3)	175.2(4)	175.4(4)	175.3(4)
**N6–N7–N8**	172.9(3)	173.8(4)	175.1(4)	174.4(4)

[a]Ref[29]

The intermolecular attractions which dictate the packing of complex **1** are shown in Figure S2 and consist of classical hydrogen bonds as well as some weak π-π and C–H···π interactions. Each molecule in the unit cell is able to participate in three intermolecular hydrogen bonds: two are established with an adjacent molecule through the interaction of the hydroxido ligand and the azide nitrogen atom (O2–H2···N3, O2–H2···N6 2.12 Å), while the third hydrogen bond interaction involves the OH group of the succinate ligand and the oxygen atom from the hydroxido belonging to a third molecule of complex (O–H5···O2, 1.706 Å). Based on the distance, both hydrogen bonds can be considered strong, but the interaction with the succinate is considerably stronger.

Additionally, the pyridine rings of complex **1** interact via two different intermolecular forces: a C–H···π interaction between the *ortho* hydrogen of a pyridine ring and the π-cloud of another pyridine ring from a neighbouring molecule (Ct1···H15, 3.025 Å), and also a very weak π-π stacking interaction between two pyridines with a centroid-centroid distance of 3.950 Å (Ct2-Ct3) (Figure S2).

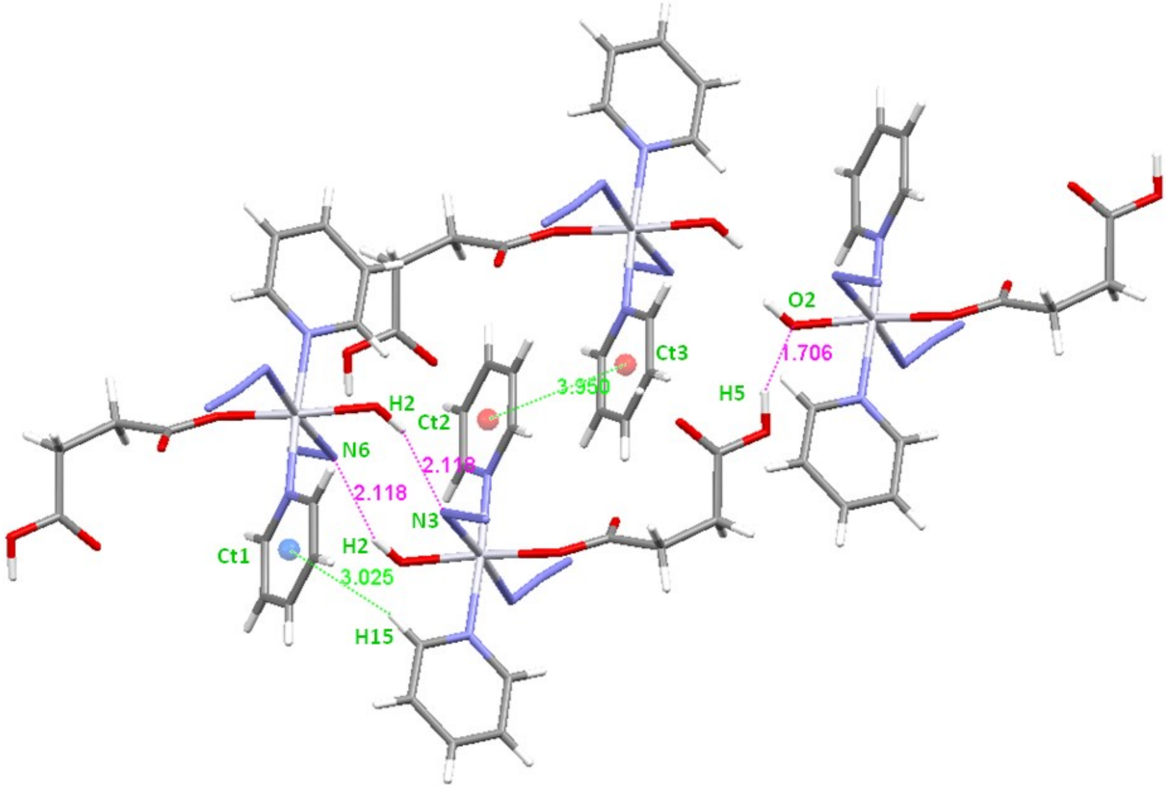

Figure S2: Intermolecular interactions in crystals of complex **1**. Classical H-bonds are denoted in pink whereas π-π interactions are shown in green. Bond distances are expressed in Å.

In complex **2** the azido ligands are not involved in any hydrogen bonding. Each molecule establishes two intermolecular hydrogen bonds between the carbonyl group of the 4-oxo-4-propoxybutanoate ligand and the hydroxido group of a nearby molecule (O2–H2···O2 1.972 Å) as shown in Figure S3. Moreover, complex **2** shows a π-π stacking interaction between pyridine rings (3.551 Å) in the crystal packing, which is stronger in comparison with that found for complex **1**.

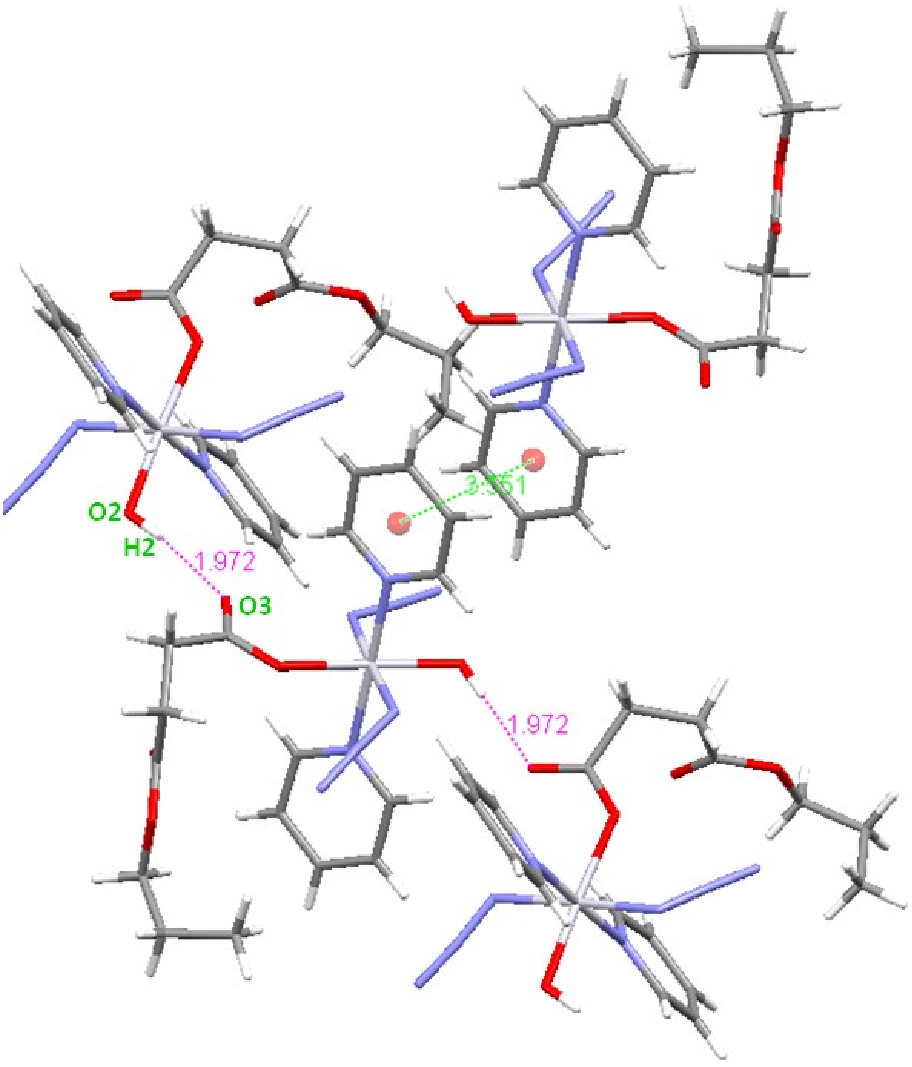

Figure S3: Intermolecular interactions of complex *trans,trans,trans*-[Pt(N_3)$_2$(OH)(succ-Pr)(py)$_2$] complex **2**. Hydrogen bond and π-π stacking interactions are denoted with purple and green colours, respectively. Bond distances are expressed in Å.

In complex **3**, each molecule in the crystallographic unit cell is hydrogen-bonded via an azido nitrogen atom (N5) and the hydroxido group (O2–H2) to another neighbouring molecule (Figure S4). The strength of this bond can be considered as medium-to-weak as it is 2.288 Å in length. The azido ligand in complex **3** which participates in an intermolecular hydrogen bond shows a weaker bond with the Pt(IV) ion than the second azido ligand does. Complex **3** is the only complex of **1**, **2** and **3** for which a strong intramolecular hydrogen bond interaction is observed. In this case the H-bond involves the carbonyl oxygen and the -NH group of the coordinated *N*-methylisatoate anion (N-H10···O3 1.096 Å), which also dictates the spatial orientation of the ligand.

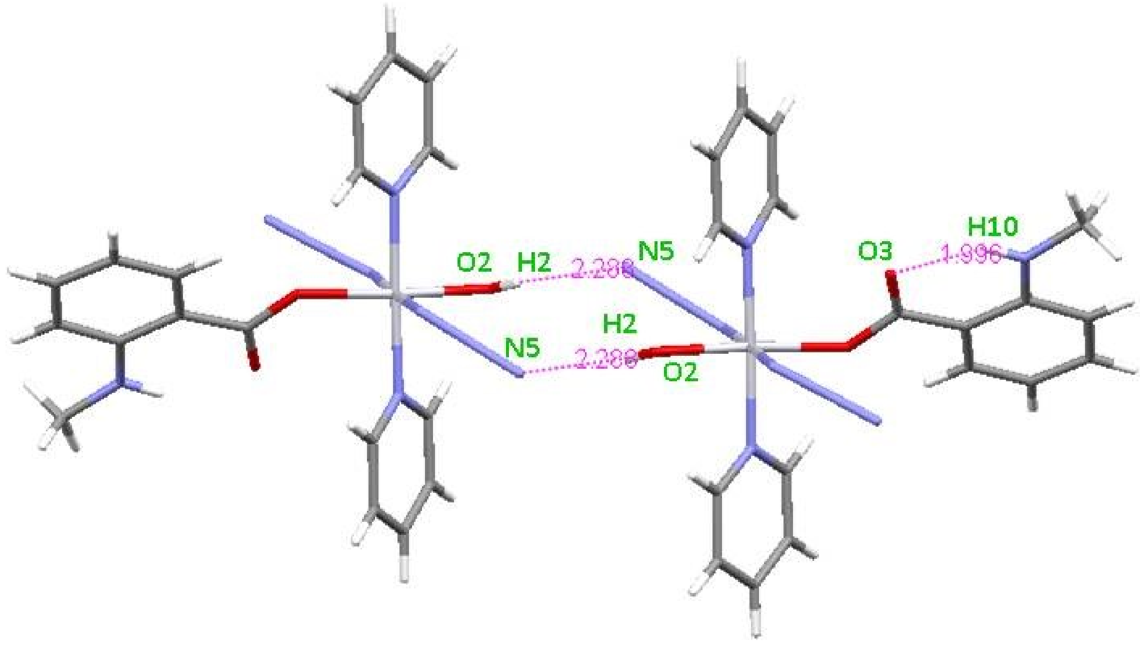

Figure S4: Intermolecular and intramolecular hydrogen bond interactions for complex *trans,trans,trans*-[Pt(N_3)$_2$(OH)(N-MI)(py)$_2$] **3**. Bond distances are expressed in Å.

pK_a determination

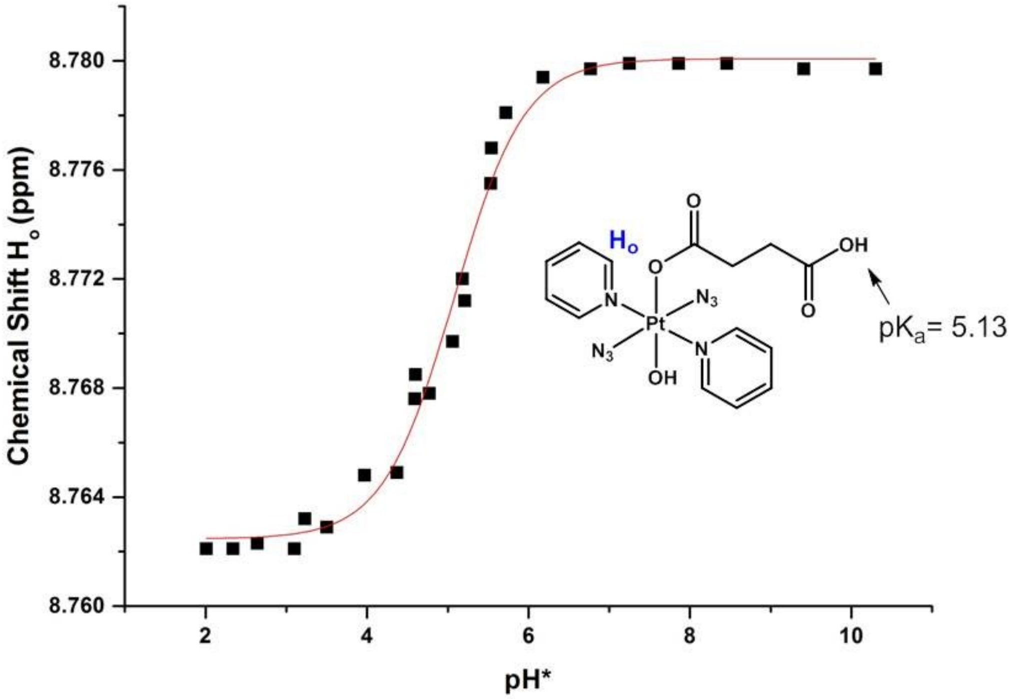

Figure S5: Change in ^{1}H NMR spectroscopic shift for the *ortho* pyridyl protons of complex **1** with respect to changes in pH*.

Extinction coefficient determination

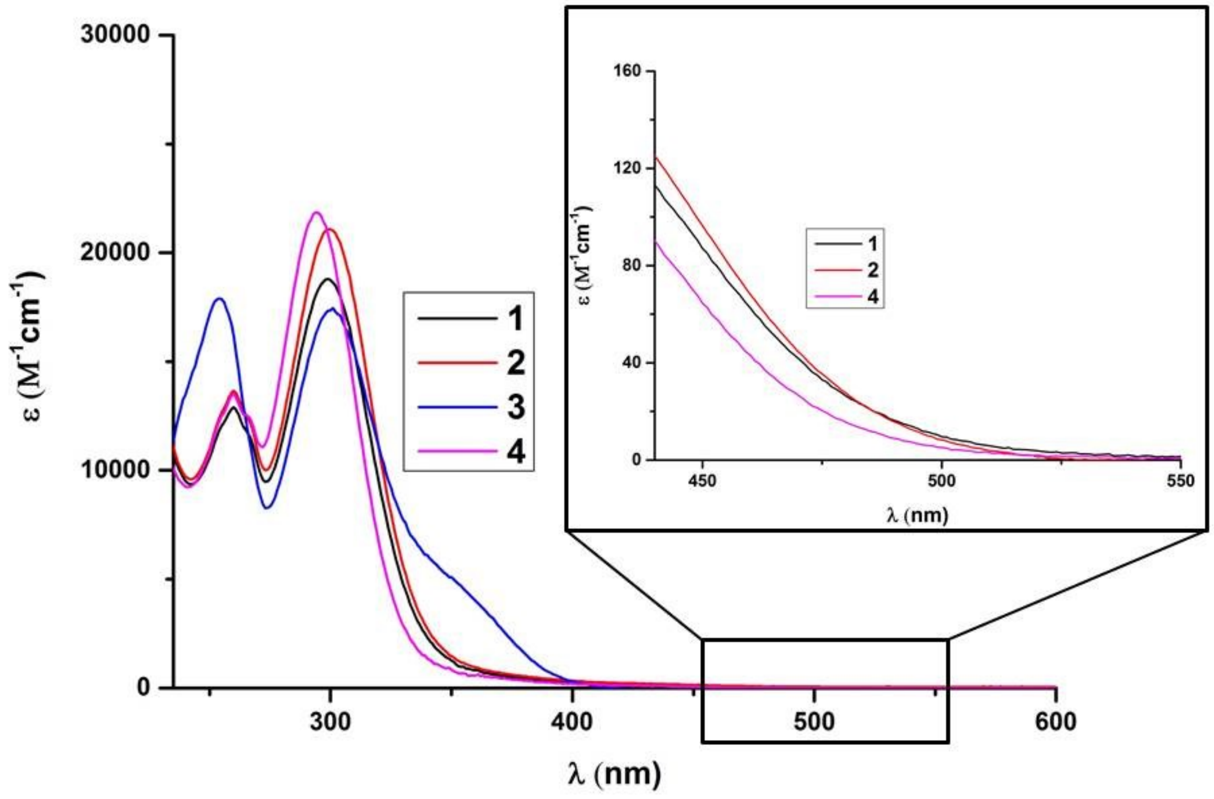

Figure S6: UV-vis spectra of complexes **1** – **3** (in H_2O for **1**, **2** and **4**; in 5% DMSO/95% H_2O for **3** due to solubility constraints). Extinction coefficients determined using ICP-MS analysis for Pt (^{194}/^{195}Pt).

DFT-TDDFT calculations

Table S3: Selected calculated (TD-DFT) singlet transitions for *trans, trans, trans*-$[Pt(N_3)_2(OH)(succ)(py)_2]$ (**1**) in the deprotonated state.

Transition	Energy (eV)	Wavelength (nm)	Oscillator Strength	Major composition
S1	2.64	469	0.0004	HOMO-3 →LUMO (63%)<br>HOMO-2 →LUMO (28%)
S2	3.00	413	0.0005	HOMO-1→LUMO (80%)<br>HOMO→LUMO (17%)
S3	3.07	404	0.0002	HOMO-1→LUMO (15%)<br>HOMO→LUMO (81%)
S4	3.13	396	0.0019	HOMO-4 → LUMO (75%)
S5	3.43	362	0.0019	HOMO-7→LUMO (28%)<br>HOMO-5→LUMO (53%)
S6	3.48	356	0.0011	HOMO-3→LUMO (30%)<br>HOMO-2→LUMO (64%)
S8	3.80	326	0.0022	HOMO-7→LUMO+2 (10%)<br>HOMO-5→LUMO+1 (10%)<br>HOMO-5→ LUMO+2 (16%)<br>HOMO-1→LUMO+1 (13%)<br>HOMO-1→LUMO+2 (10%)
S9	3.84	322	0.0018	HOMO-7→ LUMO (46%)<br>HOMO-6 →LUMO (29%)
S14	4.19	296	0.0216	HOMO-3→ LUMO+1 (34%)<br>HOMO-3→LUMO+2 (13%)<br>HOMO-2→LUMO+1 (12%)<br>HOMO-2→LUMO+2 (12%)
S19	4.31	288	0.3322	HOMO-7→ LUMO (12%)<br>HOMO-6 →LUMO (33%)<br>HOMO-5→LUMO (13%)
S22	4.437	279	0.0543	HOMO-12→LUMO (10%)<br>HOMO-11→LUMO (31%)<br>HOMO-10→ LUMO (20%)
S32	4.77	260	0.0145	HOMO-5→LUMO+1 (48%)<br>HOMO-5→LUMO+2 (31%)

Table S4: Selected Electron Difference Density Maps (EDDMS) of singlet excited state transitions for **1** in H_2O (blue indicates a decrease in electron density, while light blue indicates an increase).

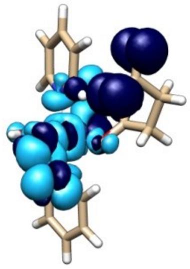 **S1**	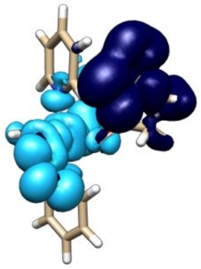 **S2**	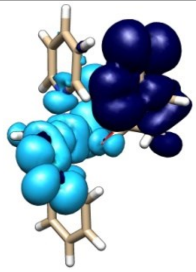 **S3**
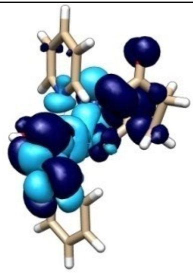 **S4**	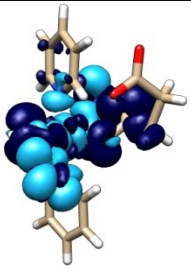 **S5**	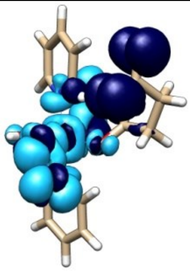 **S6**
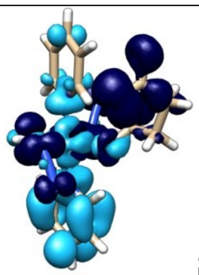 **S8**	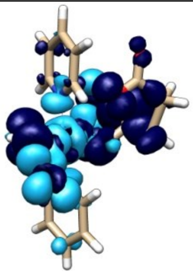 **S9**	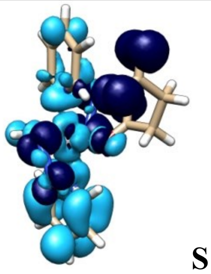 **S14**
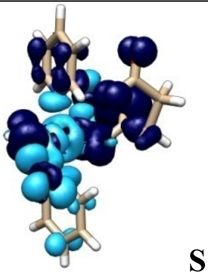 **S19**	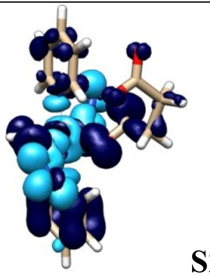 **S22**	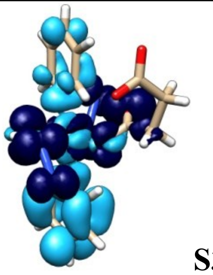 **S32**

Table S5: Selected molecular orbitals for **1** in the ground-state geometry.

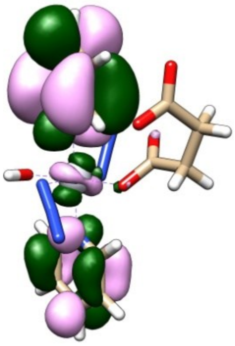 **LUMO+3**	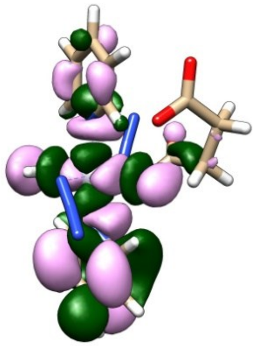 **LUMO+2**	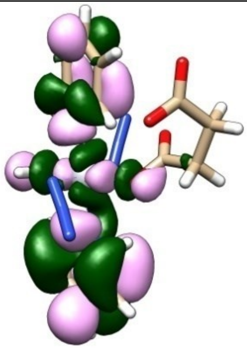 **LUMO+1**
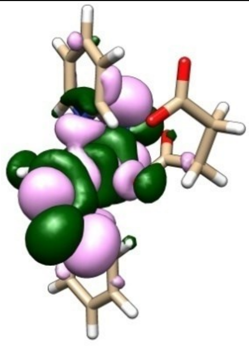 **LUMO**	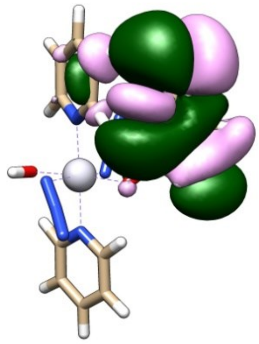 **HOMO**	
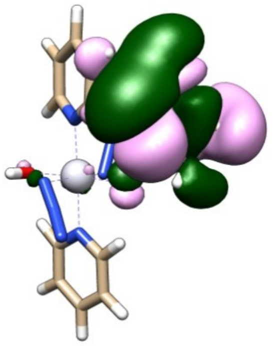 **HOMO-1**	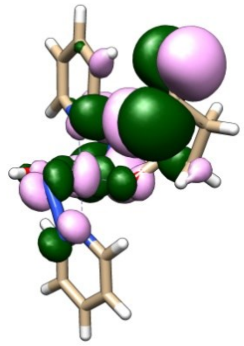 **HOMO-2**	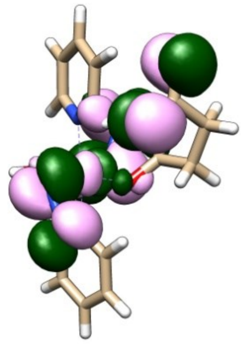 **HOMO-3**

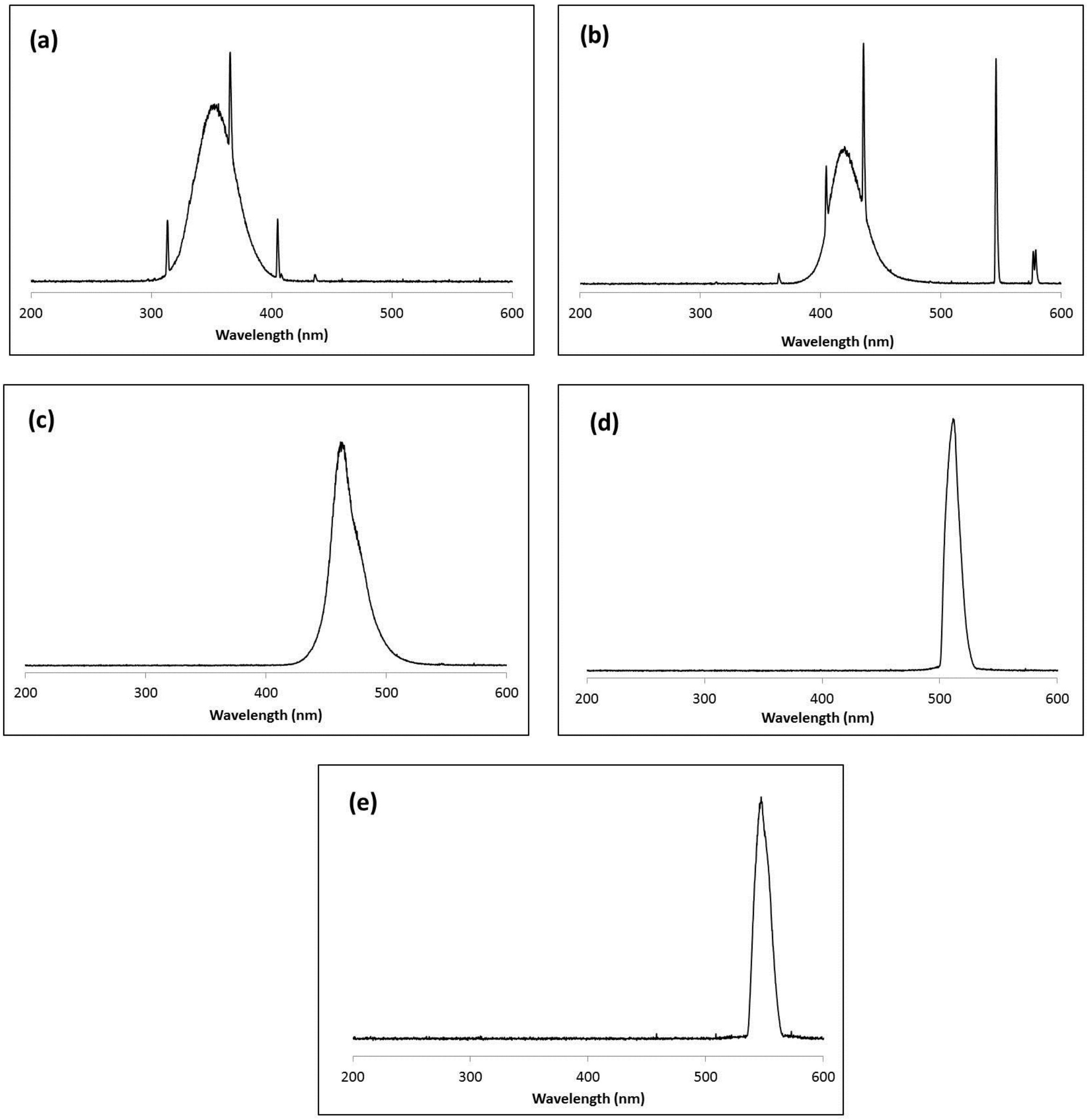

Figure S7: Output of light sources-(a) UVA lamps (Hitachi) fitted in the LZC-ICH2 photoreactor; (b) 420 nm lamps (Luzchem) fitted in the LZC-ICH2 photoreactor; (c) 463 nm, LED; (d) 517 nm, LED; (e) 550 nm KiloArc, fitted with GG530 filter, 4 nm slit widths.

Irradiation studies: UV-visible absorption spectra

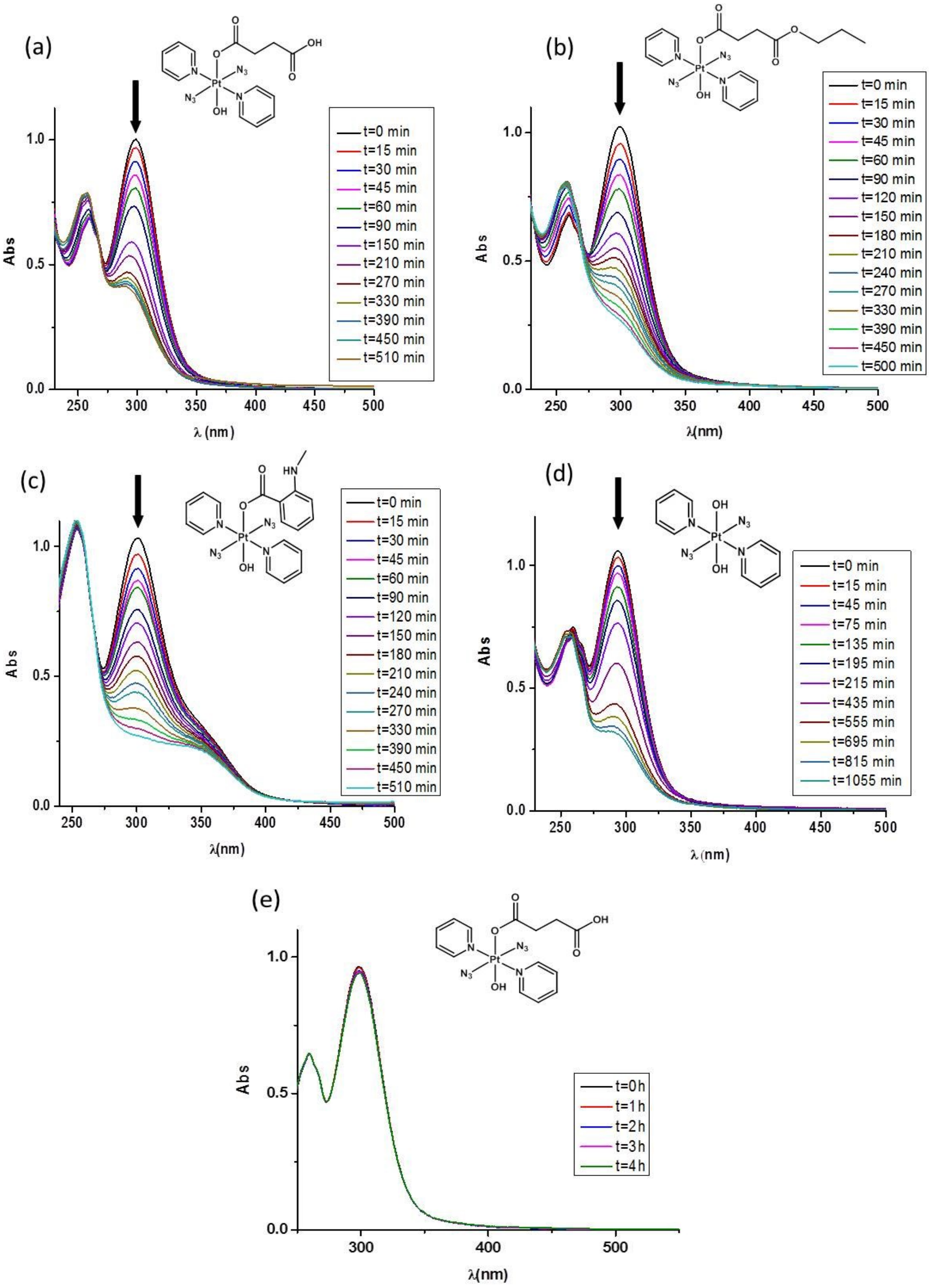

Figure S8: Changes in UV-Vis spectra of complexes **1** - **4** (a-d) (50 μM in H_2O for **1**, **2**, **4** and 95% H_2O/5% DMSO for **3**) following irradiation with green light (517 nm LED, 29 mW.cm^{-2}). e) UV-vis spectra of complex **1** following irradiation with longer-wavelength green light (550 nm, filtered below 530 nm, 4 nm slits, 2.5 mW.cm^{-2}).

Irradiation studies: NMR spectroscopy

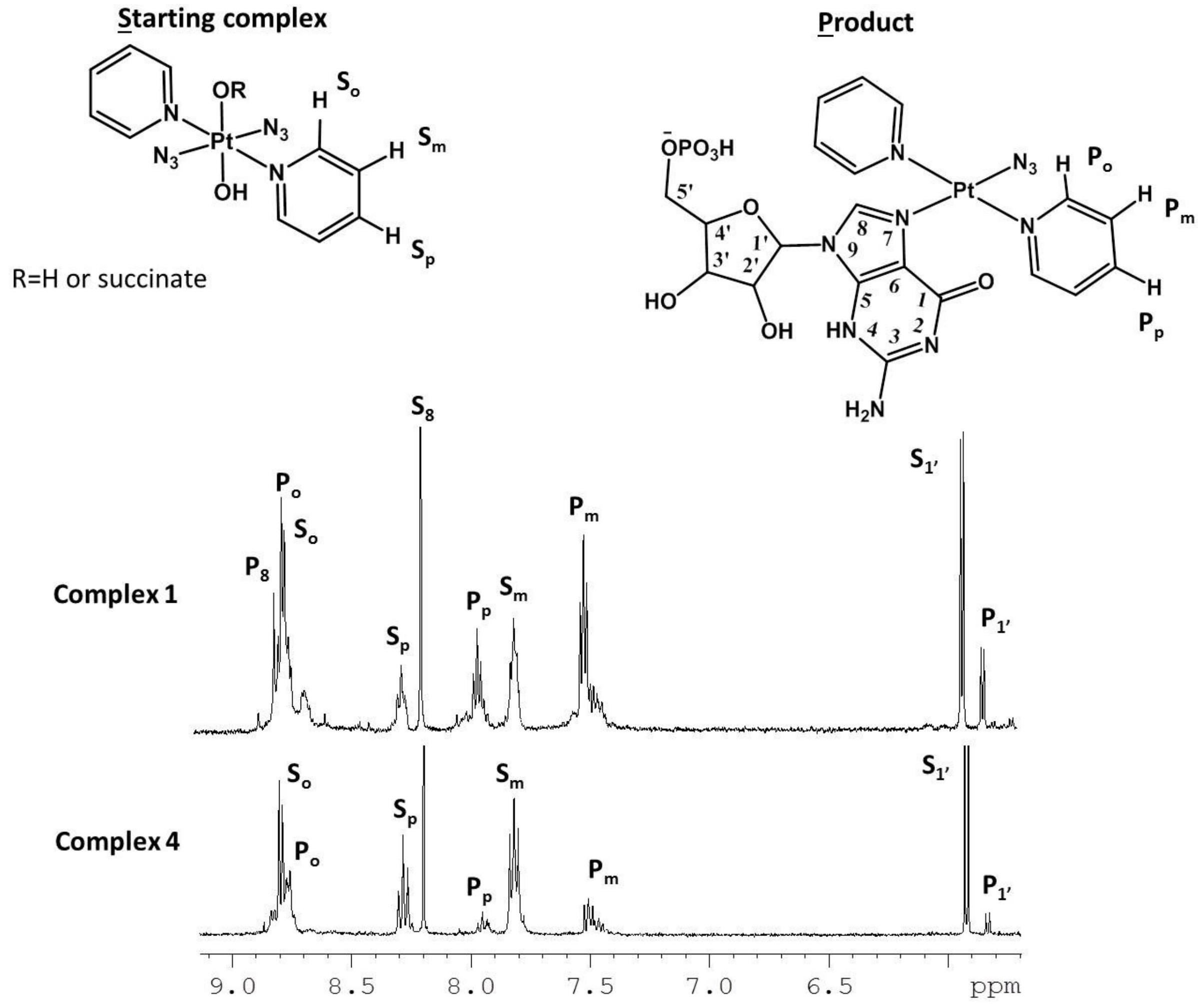

Figure S9: Comparative ^{1}H-NMR spectra (aromatic region) of complexes **1** and **4** (1.5 mM, PBS/D_2O) plus 5'-GMP (2 mol eq) after 10 min irradiation with 463 nm (64 mW.cm^{-2}) light, with the main peaks of the starting materials and products assigned.

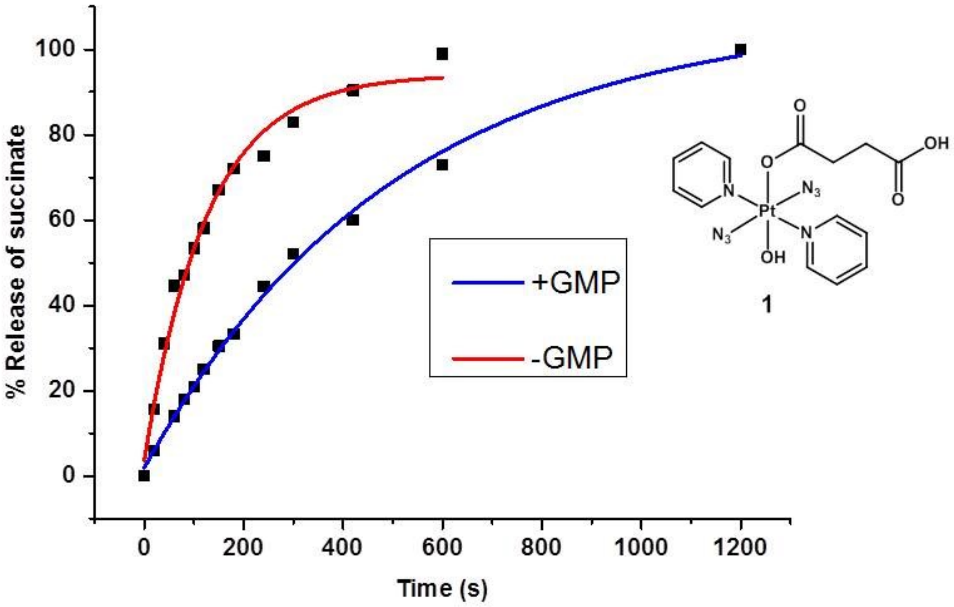

Figure S10: Time-dependent release of succinate following irradiation of **1** (1.0 mM, PBS, D_2O, pH* 7.4) with blue light (463 nm, 64 mW.cm^{-2}) in the presence (*blue line*) and absence (*red line*) of 5'-GMP (2 mol eq, 2 mM). Black dots denote the experimental data whereas the blue and red lines denote the fitted exponential function (r^2=0.98 for red and r^2= 0.996 for blue).

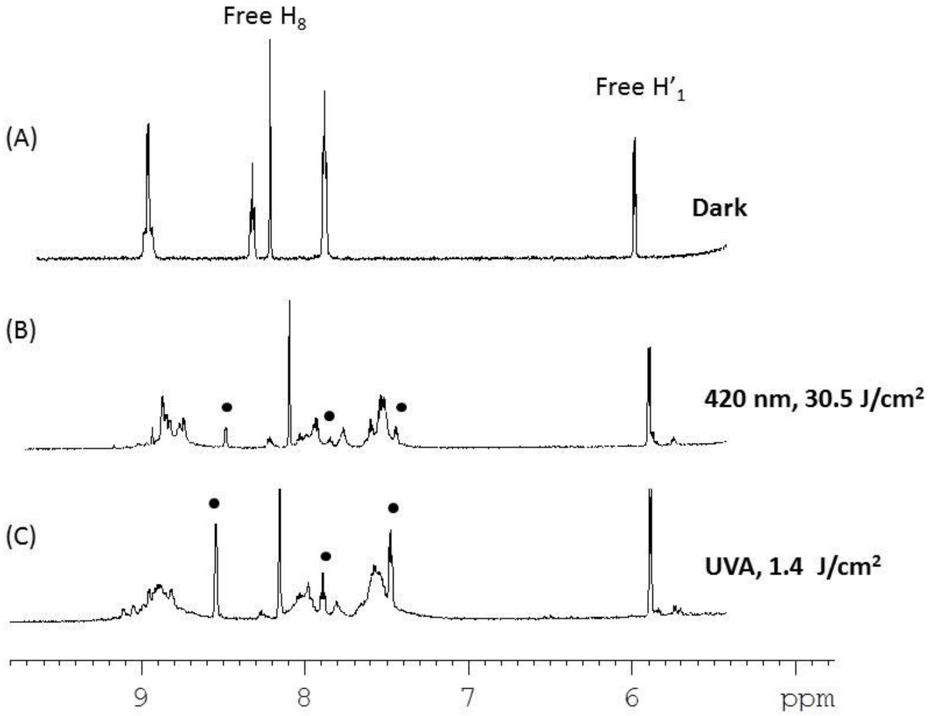

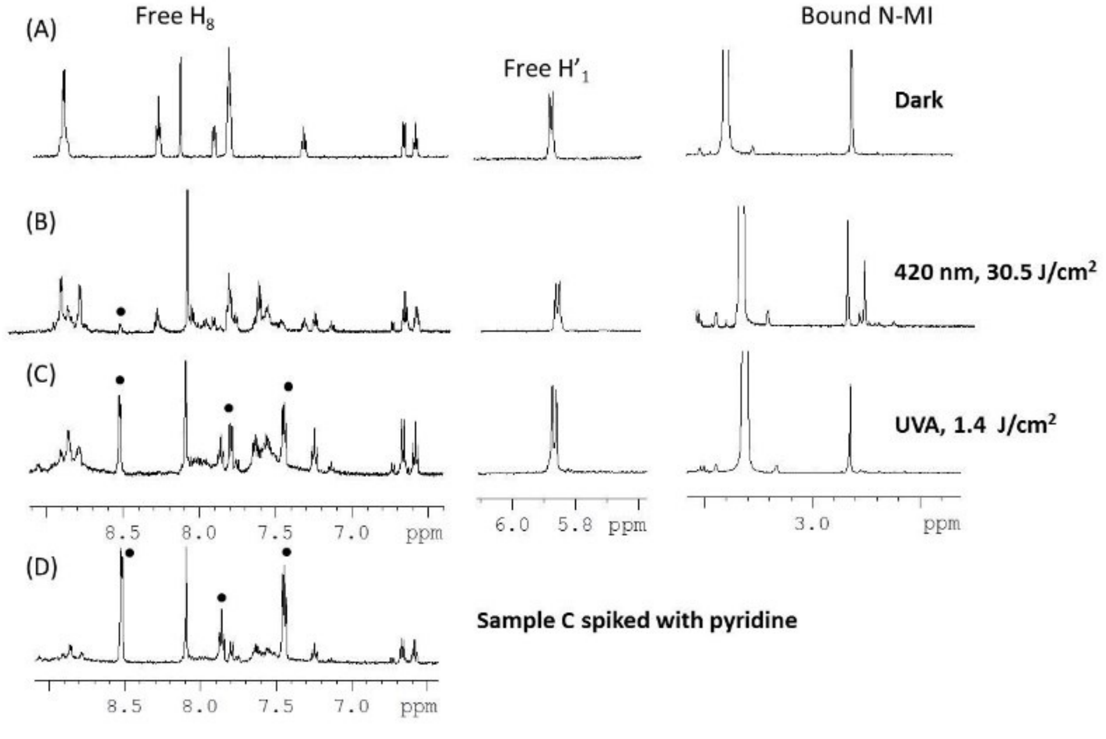

Figure S11: Top: ^{1}H-NMR spectra (500 MHz, 75% MeOH-d_4/25% D_2O (*v/v*)) of complex **3** (1.5 mM) and 5'-GMP (2 eq) (A) in the dark (with H'$_1$ of free 5'-GMP labelled) (B) following irradiation with blue light, (C) following irradiation with UVA, (D) irradiated sample spiked with pyridine. ● = released pyridine. **Bottom:** ^{1}H-NMR spectra of complex **4** (1.5 mM) and 5'-GMP (2 eq) in MeOH-d_4/D_2O 25%/75% *v/v* (A) in the dark, and under irradiation with either (B) blue or (C) UVA light.

Fluorescence measurements

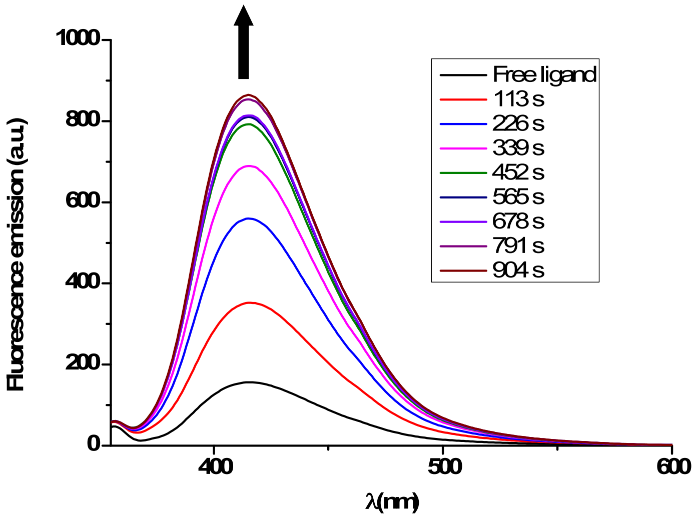

Figure S12: Fluorescence emission of the free N-MIA ligand (0.26 μM, 5% DMSO/95% H_2O) and the increase in fluorescence emission of complex **3** (26 μM, 5% DMSO/95% H_2O) upon consecutive measurements, following excitation at 320 nm. The irradiation times (in seconds) were determined by the scanning rate and the spectral range.

LC-MS analysis

Table S6: Mass spectra and putative assignment of the different species detected by LCMS after the irradiation of compound **1** (0.5 mM, λ_{irr} 420 nm, 30.5 J.cm^{-2}) in the presence of 5'-GMP (2 mol. eq.). The peak numbers correspond to the labelling in Figure 6.

Peak	Mass Spectrum	Assignment (calculated *m/z* values)
**1**	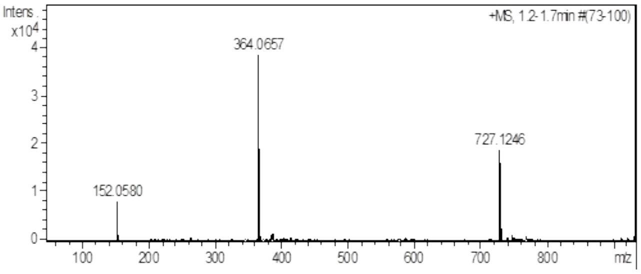	$[2GMP+H]^+$ *m/z*= 727.1246<br>$[GMP+H]^+$ *m/z*= 364.0658<br>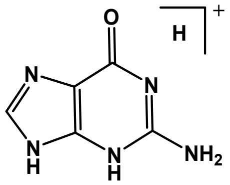<br>*m/z*= 152.0588
**2**	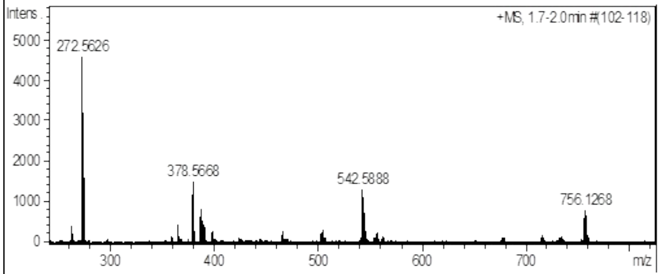	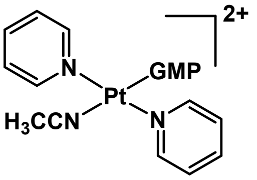<br>*m/z*= 378.5664
**3**	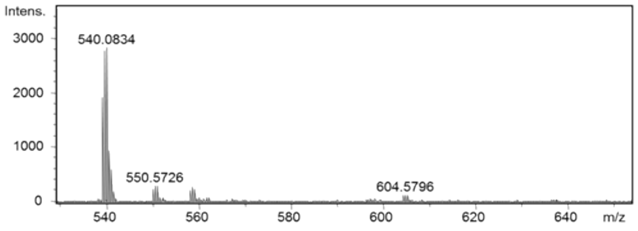	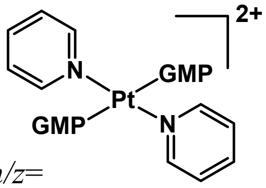<br>*m/z*= 539.5820
**5**	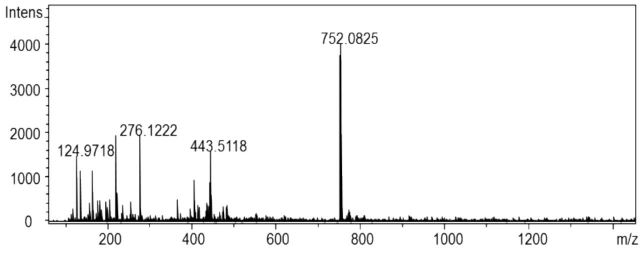	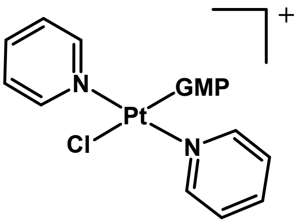<br>*m/z*= 752.0755

**6**	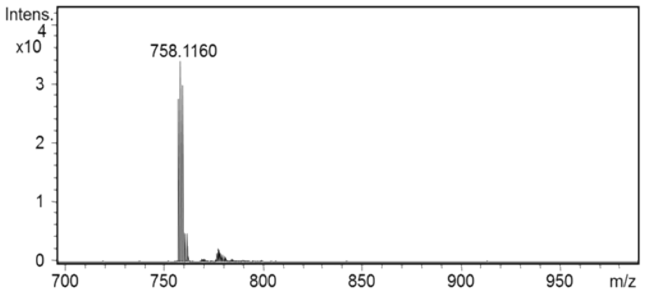	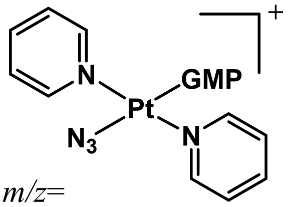 *m/z*= 758.1164
**7**	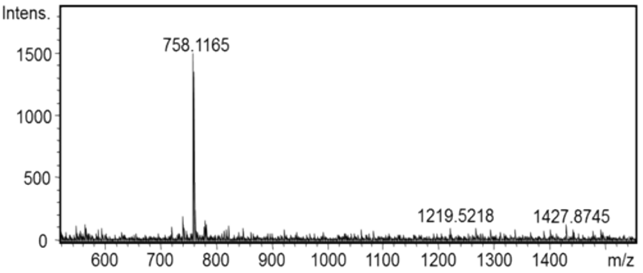	Structural isomer of mono-GMP adduct (above) *m/z*= 758.1164
**8**	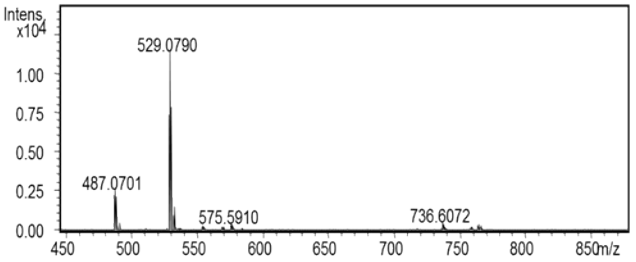	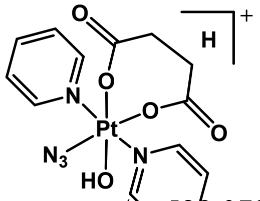 *m/z*= 529.0799<br>$[M\text{-}N_3\text{-}N_3]^+$= 487.0707
**9**	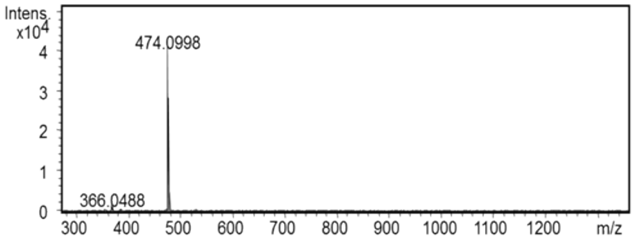	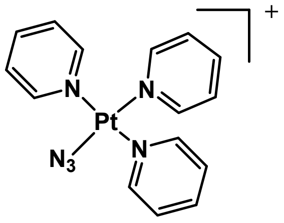 *m/z*= 474.1006
**10**	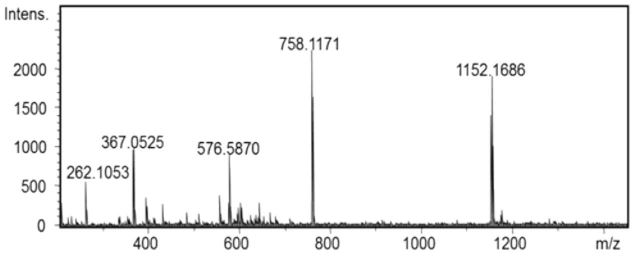	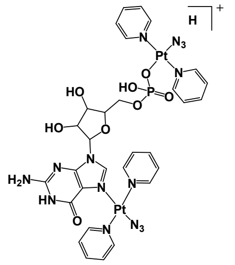 *m/z*= 1152.1727

Table S7: Mass spectra and assignment of the different species detected by LCMS after the irradiation of compound **3** (1.5 mM, λ_{irr} 420 nm, 45 min) in the presence of 5'-GMP (2 mol. eq.). The peak numbers correspond to the labelling in Figure 7.

Peak	Mass Spectrum	Assignment (calculated *m/z* values)
**1**	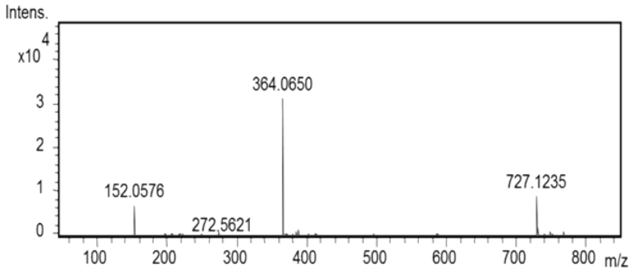	$[2GMP+H]^+$ *m/z*= 727.1246 $[GMP+H]^+$ *m/z*= 364.0658 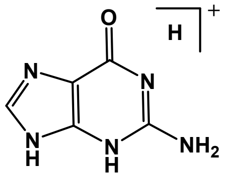 *m/z* = 152.0588
**2**	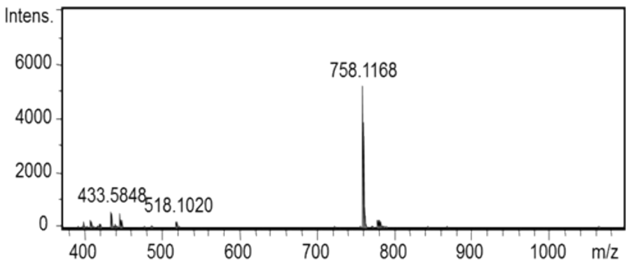	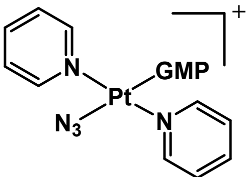 *m/z*= 758.1164
**3**	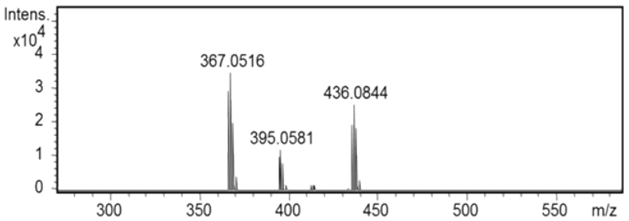	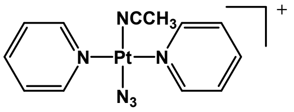 *m/z*= 436.0849 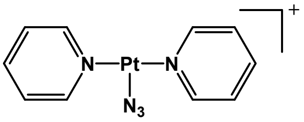 *m/z*= 395.0584 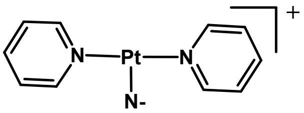 *m/z*= 367.0517

<table>
<tr><td>4</td><td>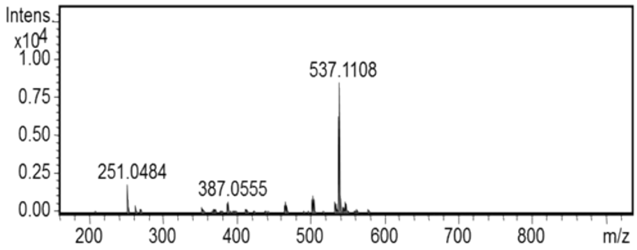
</td><td>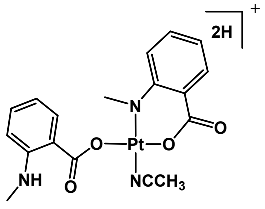

m/z= 537.1102</td></tr>
<tr><td>6</td><td>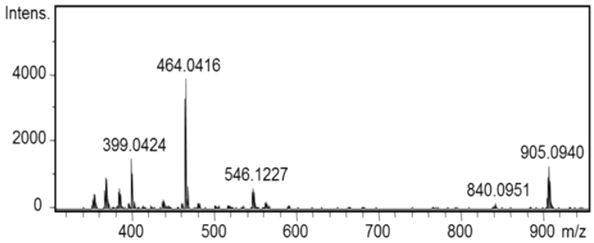
</td><td>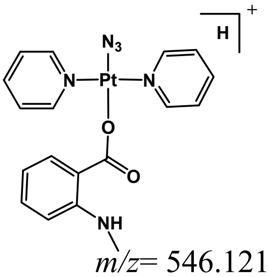

m/z= 546.1217</td></tr>
<tr><td>7</td><td>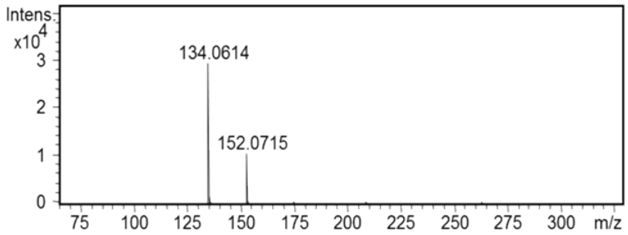
</td><td>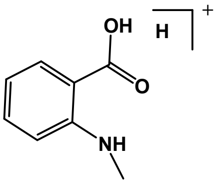

m/z= 152.0712</td></tr>
<tr><td>8</td><td>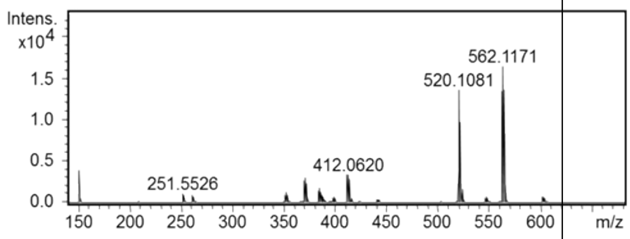
</td><td>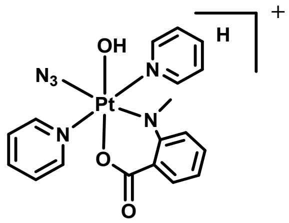

m/z= 562.1162
[M-N$_3$]$^+$</td></tr>
</table>

**9**	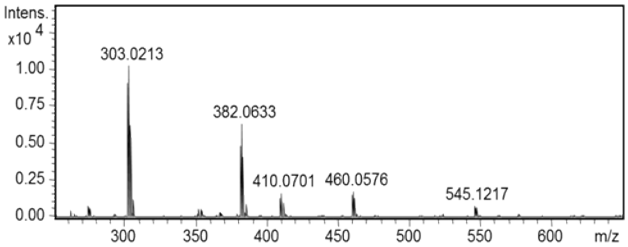	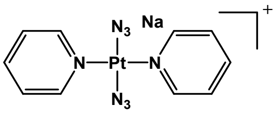<br>*m/z*= 460.0574<br>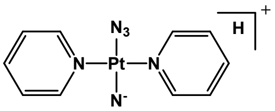<br>*m/z*= 410.0693<br>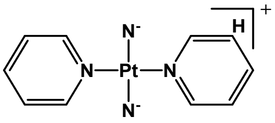<br>*m/z*= 382.0631<br>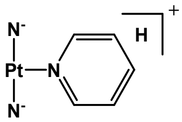<br>*m/z*= 303.0209

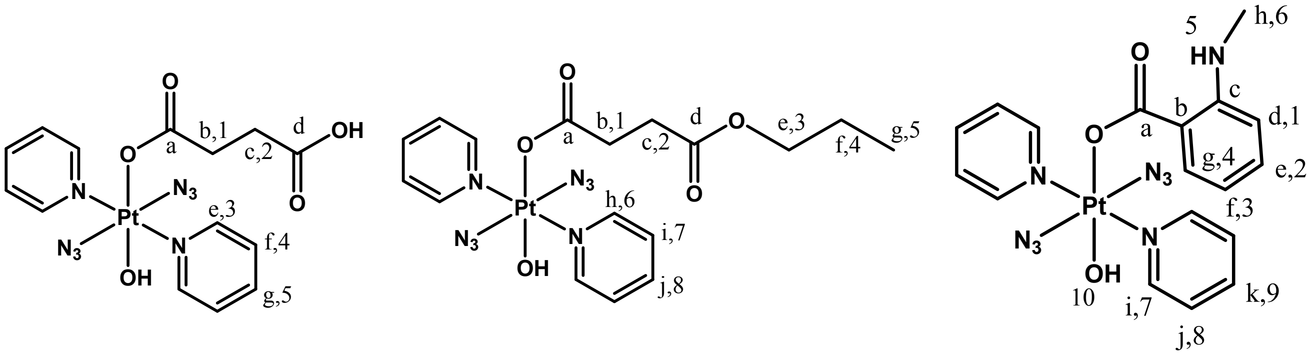

Figure S13: Numbering of hydrogens and carbons, as assigned in the experimental section.

Cell studies

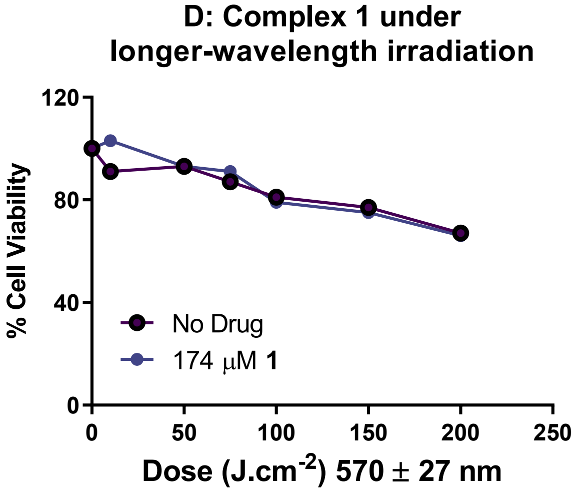

Figure S14: Anti-proliferative activity of complex **1** (175 μM) in A2780 cells under irradiation with 570 ± 27 nm (yellow) light, compared to irradiation in the absence of the complex. Data points represent the mean ± SD of one independent experiment performed in triplicate.

References

1 H. E. Gottlieb, V. Kotlyar and A. Nudelman, *J. Org. Chem.*, 1997, **3263**, 7512–7515.
2 J. W. Akitt and B. E. Mann, *NMR and Chemistry: An introduction to modern NMR spectroscopy*, Chapman & Hall, Cheltenham, 2000.
3 O. V. Dolomanov, L. J. Bourhis, R. J. Gildea, J. A. K. Howard and H. Puschmann, *J. Appl. Crystallogr.*, 2009, **42**, 339–341.
4 G. M. Sheldrick, *Acta Crystallogr. Sect. A Found. Crystallogr.*, 2008, **64**, 112–122.
5 G. M. Sheldrick, *Acta Crystallogr. Sect. C, Struct. Chem.*, 2015, **71**, 3–8.
6 A. Kręzel and W. Bal, *J. Inorg. Biochem.*, 2004, **98**, 161–166.
7 M. J. Frisch, G. W. Trucks and H. B. Schlegel, in *Gaussian 03, revision D 0.1; Gaussian Inc.: Wallingford CT.*, 2004.
8 J. . P. Perdrew, K. Burke and M. Ernzerhof, *Phys. Rev. Lett.*, 1996, **77**, 3865–3868.
9 P. J. Hay and W. R. Wadt, *J. Chem. Phys.*, 1985, **82**, 270–284.
10 A. D. McLean and G. S. Chandler, *J. Chem. Phys.*, 1980, **72**, 5639–5649.
11 M. Cossi, N. Rega, G. Scalmani and V. Barone, *J. Comput. Chem.*, 2003, **24**, 669–681.
12 M. E. Casida, C. Jamorski, K. C. Casida and D. R. Salahub, *J. Chem. Phys.*, 1998, **108**, 4439–4450.
13 R. E. Stratmann, G. E. Scuseria and M. J. Frisch, *J. Chem. Phys.*, 1998, **109**, 8218–8285.
14 F. S. Mackay, J. A. Woods, H. Moseley, J. Ferguson, A. Dawson, S. Parsons and P. J. Sadler, *Chem. Eur. J.*, 2006, **12**, 3155–3161.
15 2008, PCT/EP2007/050815.
16 N. J. Farrer, J. a. Woods, L. Salassa, Y. Zhao, K. S. Robinson, G. Clarkson, F. S. MacKay and P. J. Sadler, *Angew. Chemie-International Ed.*, 2010, **49**, 8905–8908.
